# Methylation Mesa define functional regulatory elements for targeted gene activation

**DOI:** 10.21203/rs.3.rs-4359582/v1

**Published:** 2024-10-16

**Authors:** Y.V. Liu, J. Suryatenggara, H. Wong, M.K. Jayasinghe, J.P. Tang, H.K. Tan, J. Kwon, Q. Zhou, S. Ummarino, A.K. Ebralidze, M.T.N. Le, J.G. Doench, L. Chai, T. Benoukraf, D. Hiwase, D. Thomas, A. Di Ruscio, D.G. Tenen, M.A. Bassal

**Affiliations:** 1 Cancer Science Institute of Singapore, 117599, Singapore; 2 Genetic Perturbation Platform, Broad Institute, Cambridge, MA 02142, USA; 3 Department of Pharmacology, Yong Loo Lin School of Medicine, National University of Singapore, 119077, Singapore; 4 Harvard Stem Cell Institute, Harvard Medical School, Boston, MA 02115 USA; 5 Department of Pathology, Brigham and Women’s Hospital, Boston, MA 02115, USA; 6 Discipline of Genetics, Faculty of Medicine, Memorial University of Newfoundland, St. John’s, NL, A1B 3V6, Canada;; 7 Royal Adelaide Hospital, Central Adelaide Local Health Network, Adelaide, South Australia;; 8 Precision Medicine Theme, South Australian Health and Medical Research Institute, Adelaide, South Australia; 9 Adelaide Medical School, The University of Adelaide, Adelaide, South Australia; 10 Department of Translational Medicine, University of Eastern Piedmont, Novara, 28100, Italy; 11 Harvard Medical School Initiative for RNA Medicine, Harvard Medical School, Boston, MA, 02115, USA; 12 Cancer Research Institute, Beth Israel Deaconess Medical Center, Boston, 330 Brookline Avenue Boston, MA 02215

**Keywords:** DNA Methylation, Demethylation, Methylation Sensitive Sites, CRISPR-DiR, Gene Activation, Gene Expression

## Abstract

DNA methylation and mRNA expression correlations are often presented with inconsistent evidence supporting causal regulation. We hypothesized that causal regulatory methylation elements would exhibit heightened demethylation sensitivity. To investigate, we analyzed 20 whole-genomic bisulfite sequenced samples before and after demethylation and identified narrow-width (45–294 bp) elements within a short plateau, termed Methylation Mesa (MM). The Mesa signature was conserved across species and was independent of CpG islands. Mesa also demonstrate high concordance with primed and active histone marks. To assess causality, we developed CRISPR-DiR, a highly precise targeted demethylation technology. Targeted demethylation of a Mesa triggers locus and distal chromatin rewiring events that initiate mRNA expression significantly greater than promoter-CpG island targeting. Thus, Mesa are self-sustaining epigenetic regulatory elements that maintain long-term gene activation through focused demethylation only within the Mesa core, resulting in subsequent histone modifications and chromatin rewiring events that interact with distal elements also marked as Mesas.

## Introduction

DNA methylation is an epigenetic mechanism capable of transmitting information and regulating gene activity without modifying nucleotide sequence^1^. Aberrant DNA methylation, in regions such as gene promoters, is considered an epigenetic hallmark of many cancers, contributing to tumorigenesis by means of transcriptionally silencing tumor-suppressor genes^2–4^. For decades, studies have endeavored to identify functionally important methylation regulatory elements that correlate with gene expression, as these would be of significance in understanding disease development mechanisms, as diagnostic biomarkers, or as prime targets for targeted epigenetic therapies.

Cytosine methylation within CpG dinucleotides represents the dominant DNA methylation event in human somatic cells. Investigations have shown that CpG dinucleotides are enriched in CpG island (CGI) clusters where, in humans, 40%–70% of gene promoters overlap with a CGI^5–9^ (depending on genome build and CGI selection criteria). Promoter-CGIs, generally unmethylated in normal cells but abnormally methylated in cancers, are the most widely accepted regulatory elements whose aberrant high methylation contributes to gene silencing^3^, even though evidence has been presented that at least some of the remaining 30–60% of non-CGI genes also show methylation-dependent transcriptional regulation^10,11^. For decades, methylation changes in promoter-CGIs have been the focus of developed cancer biomarkers for diagnosis and response to therapy with limited clinical success^12^. In fact, many studies have reported contrary evidence with inconsistent or weak associations between promoter-CGI methylation and gene expression^13–15^. Advancements in technology have allowed for more comprehensive methylome profiling, revealing more dynamic methylation destinations beyond CGIs, such as CpG shores^15^. These regions have been described as demonstrating a stronger correlation with transcription, suggesting a more significant role than previously understood, even though most evidence remains associative rather than causal; i.e., while hyper-methylated loci are demonstrated to associate with gene silencing, the converse, demethylation and causal re-expression, has not been readily demonstrated in most cases. Although compelling, these and other emerging destinations are defined in “kilobase” (kb) sizes and show variable tissue specificity, thus limiting their accuracy to serve as guidelines for narrow and precise causal methylation-sensitive regulatory elements. This is exemplified by the reports that methylation-sensitive “core” regions showing better correlation with transcription have been characterized both within and outside of upstream promoter-CGI^16,17^, indicating the existence of methylation regulated causal elements that have not been precisely defined and annotated. However, instead of searching for said causal demethylation regions, most studies extensively focus on either computationally predicted gene promoters (up to 2kb upstream of the TSS) or CGI, thus leading to an aforementioned question regarding the precise location of regulatory elements.

With the development of targeted epigenetic modification technologies, such as CRISPR DNA-demethylation tool dCas9-TET1^18,19^, it is now possible to initiate DNA demethylation in a locus-specific manner. However, due to the nature of the fused functional proteins, as well as the structural design of these technologies, such epigenetic modification systems tend to have wide editing windows^18,19^, limiting their resolution and accuracy to pinpoint causal methylation regulatory elements of interest.

In this study, we hypothesized that methylation-sensitive regulatory elements with causal functional impact should possess heightened sensitivity to demethylation stimuli. So, we employed whole-genomic bisulfite sequencing (WGBS) to comprehensively profile 20 primary patient and cell line samples before and after hypomethylating agent (HMA) treatment. Following analysis of over 3.2 terabytes of WGBS data, we identified a uniform, narrow-width methylation signature throughout the genome, independent of CGI, consistent across both human and murine cell lines and primary patient samples. We designated this signature as Methylation Mesa (MM). By utilizing matched bulk RNA-seq data where available, we observed a stronger link between 5’UTR/Exon1 Mesa demethylation and transcriptional activation than with upstream-TSS promoters. To directly validate the causal regulatory nature of Mesa, we developed a high-resolution targeted demethylation technology, CRISPR-DiR, with a 10 times narrower demethylation window compared to existing tools such as CRISPR-TET1. Utilizing the hypermethylated tumor suppressor CDKN2A (p16^INK4a^) as a model, we observed that CRISPR-DiR targeted demethylation of TSS-proximal Mesas resulted in higher gene re-expression than targeting the upstream promoter-CGI. Furthermore, chromatin remodeling consequent to p16 TSS-proximal Mesa demethylation resulted in the formation of looping interactions between p16 TSS-proximal and distal Mesas.

In summary, we have identified MM as causal DNA methylation regulatory elements identifiable genome-wide and used CRISPR-DiR, a fine-resolution targeted demethylation technology, to demonstrate their causative role in gene activation. These findings pave the way for providing insights into diseases characterized by abnormal DNA methylation patterns, enabling the identification of novel biomarkers and potential targeted epigenetic therapies.

## Results

### A distinct and narrow-width Mesa signature is consistent across sample types and species.

We hypothesized that methylation-sensitive regulatory seed elements would possess a heightened sensitivity to demethylation stimuli than flanking loci. Although DNA hypomethylating agents (HMA), such as Azacytidine (AZA) and Decitabine (DAC), are regarded as nonspecific agents with biologically differing modes of action^20,21^, their observed effects on the genome are not uniformly distributed^22^, suggesting a heightened sensitivity of particular loci to their effects. We therefore opted to deeply profile the methylation changes of paired samples before and after HMA treatment to detect methylation hyper-sensitive sites. In total, whole-genomic bisulfite sequencing (WGBS) was performed on 20 paired samples (pre/post-HMA treatment; generated in-house or obtained from public repositories) across a breadth of cell types with diverse genetic backgrounds ranging from human to mouse, including 4 (pre-)leukemia patients (2 Chronic Myelomonocytic Leukemia (CMML), 1 Refractory anemia with excess blasts-Myelodysplastic Syndromes (RAEB-MDS), 1 Acute Myeloid Leukemia (AML)), 15 human cancer line samples from 7 cell types (SNU398, HCT116, K562, KG1a, MDS-L, MOLM13, MOLM14), 1 PDX, and 1 mouse line (CT26). Furthermore, we utilized a DNMT1 knockout HCT116 line to gain insight into which loci require methylation maintenance to remain hypermethylated in the naïve state.

**Figure 1.**
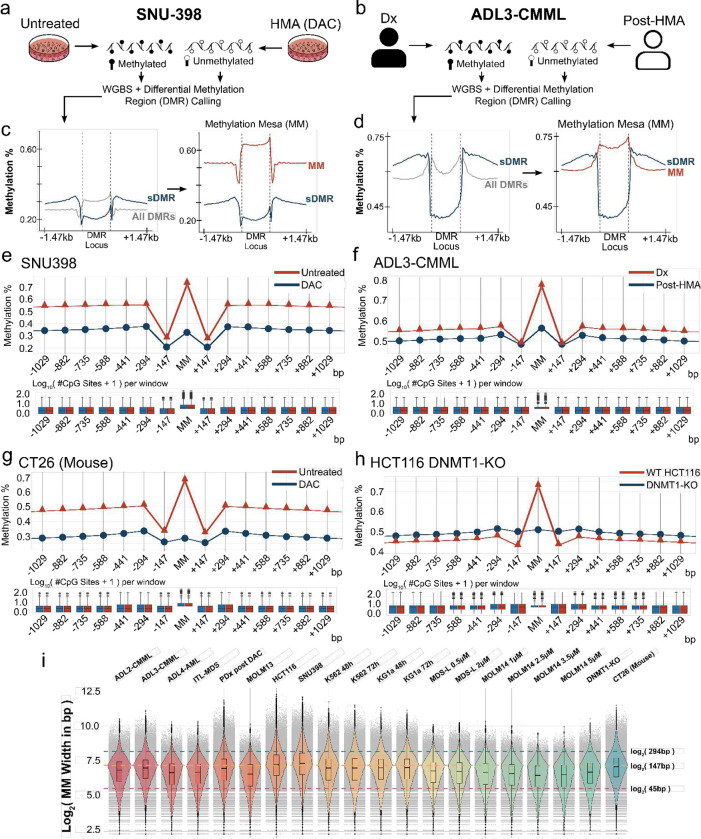
Characterization of Methylation Mesa signatures following hypomethylating agent (HMA) induced demethylation (**a**) Schematic diagram depicting how cell lines were handled using SNU398 as an example. Naïve/untreated and HMA (DAC) treatment cells were subjected to WGBS and differentially methylated regions (DMRs) called (see **Methods**). (**b**) Same as (**a**) but showing the ADL-CMML patient derived samples. Samples were collected at diagnosis (Dx) and after therapy (Post-HMA), then subjected to WGBS and DMR calling. (**c,d**) DMR’s were called using HMA-treated against untreated/naïve controls. Within all the identified DMR’s (grey), a subset of regions can be identified which show hyper-sensitivity to demethylation stimuli as compared to their flanking loci (sDMRs; blue lines). When observing the methylation profile of the same region, but in the naïve/untreated samples, a distinct profile (red) is observed. The Y-axis shows methylation percent ranging from 0 (no methylation, 0%) to 1 (methylated, 100%). The X-axis shows the DMR locus between the two dashed lines in the center. Extending bidirectionally, are methylation curves up to 1.47kb from the edge of the respective DMR locus in each direction (up- and downstream). (**e,f,g,h**) Average methylation profiles for untreated and HMA-treated samples showing Mesa and flanking window methylation percentages. Below the methylation profiles are matched boxplots depicting the distribution of the number of CpG sites found per corresponding window. The Y-axis shows methylation value ranging from 0 (no methylation, 0%) to 1 (methylated, 100%). The X-axis shows the MM in the center, and extending bidirectionally from each MM are the flanking windows, each 147bp in length, denoted by their relative base pair distance away from the Mesa. Within each group along the X-axis, the profiles plotted show the average methylation value across all sites identified genome-wide within that window distance away from the Mesa. The profiles corresponding to untreated/naïve samples are shown in red. The profiles corresponding to post-HMA treatment are shown in blue. Below each average methylation profile are box plots summarising the distribution of the CpG sites (log_10_ (# CpG’s +1)) profiled within that window. Red box plots represent summaries of the number of CpGs found in the untreated/naïve samples, while the blue box plots show summaries of the number of CpG sites profiled in the HMA-treated sample. (**e**) Herein the average methylation profile for untreated and DAC treated samples are shown for the SNU-398 line. (**f**) Herein we show the average methylation profile for Dx and post-HMA treatment of the ADL3-CMML patient sample. (**g**) Herein we show the average methylation profiles for a publicly available and re-processed CT26 mouse cell line sample. Supplemental Figure 1 shows all the remaining average methylation profiles in this study. (**h**) The Y-axis shows methylation value ranging from 0 (no methylation, 0%) to 1 (methylated, 100%). The X-axis shows the MM in the center, and extending bidirectionally from each MM are the flanking windows, each 147bp in length, denoted by their relative base pair distance away from the Mesa. Within each group along the X-axis, the profiles plotted show the average methylation value of that window across all sites identified genome-wide. Shown in red is methylation profile observable in a naïve HCT116 line. Shown in blue is the methylation profile of the untreated HCT116 DNMT1^−/−^-KO sample. (**i**) Violin plot showing the distribution of Mesa sizes across all profiled samples. The X-axis shows all samples profiled in this study. The Y-axis shows the (log_2_) size of all MM (in bp) identified in the respective sample. All samples had Mesa sites with sizes predominantly between 45–294 bp.

For cell lines, samples were cultured with Azacytidine/Decitabine and compared to untreated DMSO control (see **Methods** and a representative figure in Figure 1a). For patients, diagnostic (Dx) samples were compared to post-HMA treatment (see **Methods**, representative figure in Figure 1b). Our analysis defined differentially methylated regions (DMR’s) as showing at least a 10% reduction in methylation when called across matched sample pairs (pre/post-HMA, pre/post-DNMT1KO; Figure 1c, d (grey)). Within all DMRs, we hypothesized that core methylation-sensitive regulatory seeds should show greater demethylation sensitivity than their bi-directional flanks post-HMA treatment or DNMT1 depletion. To identify, we divided each DMR flanking region into small, biologically associated length windows, hypothesizing that functional methylation changes would revolve around nucleosome length units, thus searching flanking loci in 147 bp length windows. We therefore calculated methylation across ten windows (147 bp*10, 1470 bp total) on each side of a DMR, therefore making our total search comparable to windows frequently used in published studies (1–2Kb). We then filtered for seed DMRs that showed at least a 10% lower average methylation than *both* bidirectional flanks. Diagrammatically, such hyper-sensitivity appears as a “high-low-high” or “smiling” DMR (sDMR) profile (representative figures in Figure 1c, d (blue)). When observing the average methylation profiles of the same sDMR locus but in naïve cells, we consistently observed a strong methylation signal that is bidirectionally flanked by lower methylation arms (representative figures in Figure 1c, d (red)). We thus named such sites showing the highest methylation in the naïve state, which switches to a “smiling” DMR (sDMR) profile following a demethylation trigger as a **M**ethylation **M**esa (MM), as the methylation profile resembles the geographical rock formation of the same name.

To better understand the methylation profiles surrounding Mesas, we plotted the average methylation of each Mesa and each flanking window. Across all profiled samples, we saw consistent, near identical MM and flanking profiles across all sample pairs (Figure 1e–g, Supplemental Figure 1). We also observed a consistent methylation buffering window at ~294–441 bp (equivalent to ~2–3 nucleosomes width away from the MM) that appears to serve as a boundary demarcating a Mesa locus from surrounding regions. This signature was observed across all samples and across species, signifying a conserved epigenetic feature rather than a sample dependent observation (Figure 1e–g, Supplemental Figure 1).

In order to gain an insight into how Mesa methylation is regulated, we sequenced an HCT116 DNMT1^−/−^-KO line and compared it to the naïve HCT116 parental line which revealed that mesa methylation maintenance appears to require DNMT1. In the absence of DNMT1, the methylation profile across the genome lacked the characteristic Mesa methylation peak typically seen in naïve states (Figure 1h).

In order to ensure the identified Mesa profiles were not skewed by, or are a consequence of disparate CpG densities, we calculated the number of CpG sites per window. We noted that, on average, no significant differences across each window were observed (Figure 1e–h, Supplemental Figure 1, box-plots beneath profiles). Finally, when ascertaining the length distributions of Mesas, we observed a consistent trend across all 20 sample pairs of narrow-width elements predominantly between 45–294 bp (Figure 1i). This is consistent with our hypothesis that causal regulatory seeds should be exiguous.

In summary, high-resolution methylation profiling of both naïve and demethylated sample pairs across both human and mouse samples identified narrow-width MM, genomic loci with heightened sensitivity to demethylation stimuli which, in naïve cell states, have a methylation profile resembling a geographical Mesa formation with bidirectional flanking boundaries of ~294–441 bp. This previously un-annotated epigenomic profile appears to be independent of the sample used, as it was observed nearly identically in both human and murine cells. These sites also appear to show methylation maintenance by DNMT1.

### Mesas enrich in 5’UTR and better correlate with gene activation than upstream promoters

**Figure 2.**
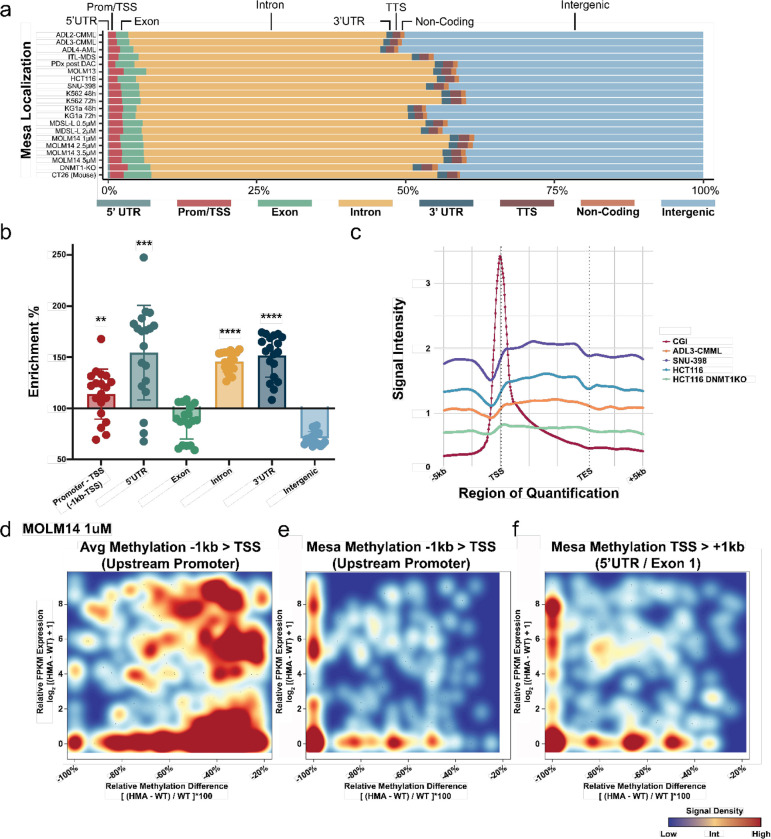
Characterization of MM across genomic localizations (**a**) Cumulative bar graph of Mesa percentages across genomic localizations. The cumulative bar graph shows the distribution of Mesa sites across all identified genomic localizations totalling 100% for all Mesa genome-wide. All samples showed relatively consistent distributions of Mesas across all compartments. (**b**) Mesa enrichment per category. Mesa percentages per functional category were normalized to genomic baseline distributions. Shown on the Y-axis is the enrichment percent as compared to genomic baseline. The X-axis shows the annotated localizations. Each dot is a sample profiled in this study (listed in Figure 2a). Enrichments > 100% indicate an enrichment greater than genomic baseline. Enrichments < 100% denote enrichment less than genomic baseline. (**c**) Profile plot showing presence of signal surrounding coding genes for selected samples. Along the region of −5kb > TSS > TES > +5kb surrounding all coding genes, 50bp bins were created and the presence (signal) of a CGI or Mesa was calculated. The profile plot shows the signal across all 50bp bins with a loess smoothing filter applied. The profile presented shows peak CGI signal just upstream of gene TSS, while Mesa elements conversely associate with CGI and instead peak within gene bodies (enriched in 5’UTRs and introns as shown in Figure 2b). (**d-f**) Density plots showing the association between methylation and mRNA expression. Only genes for which there was methylation and mRNA signal across all 3 compartments were utilized so as to provide consistent comparison. The X-axis shows the relative methylation difference percent between HMA-treatment and untreated/naïve (i.e., what percent does the methylation drop in the HMA-treated sample as compared to the methylation level observed in the untreated/naïve sample). The Y-axis shows the relative FPKM expression difference between HMA-treatment and untreated/naïve (i.e., how much does the expression change in the HMA-treated sample with respect to the untreated/naïve sample). (**d**) Density plot showing the association between average methylation across the entire 1kb promoter upstream of a TSS and mRNA expression. Signal density shows many genes with low relative methylation difference (i.e., there is little methylation difference between HMA-treatment and untreated/naïve) yet still have high relative expression, indicating a weak association between methylation level and expression. (**e**) Density plot showing the association between average methylation of just Mesa loci within the 1kb promoter upstream of a TSS and mRNA expression. Signal density shows (along the X-axis), genes that are undergoing demethylation (demethylating from −20 > −100%) but have still not shown expression (y=0) with most genes showing increases in relative expression only after achieving near −100% relative methylation drop in the HMA-treated sample as compared to the untreated/naïve. (**f**) Density plot showing the association between average methylation of just Mesa loci located within the first 1kb of a gene body (i.e., covering a gene’s 5’UTR and exon 1) and mRNA expression. Signal density shows (along the X-axis), genes that are undergoing demethylation (demethylating from −20 > −100%) but have still not shown expression (y=0) with most genes showing increases in relative expression only after achieving near −100% relative methylation drop in the HMA-treated sample as compared to the untreated/naïve. Panel (**f**) differs from panel (**e**) in that it shows a greater density of genes with high relative expression difference and complete relative methylation difference (i.e., you get a better associative relationship between methylation of 5’UTR/exon1 Mesas and expression than by associating methylation of Mesas in the 1kb promoter region with expression).

We next investigated the genomic distribution of Mesas. Using RefSeq defined genomic features (assembly GCA_000001405.28, release 107), we annotated the localization of Mesas and observed consistent genome-wide distributions across all major functional categories, across all samples (Figure 2a). To determine enrichment, we normalized Mesa percentages in each feature category against the natural genome percentage of the corresponding category. Doing so revealed that Mesas were found most enriched in gene 5’UTRs (155% enrichment, p = 6*10^−4^, Bonferroni adjusted), followed by 3’UTRs (151% enrichment, p < 1*10^−15^ Bonferroni adjusted), introns (145% enrichment, p < 1*10^−15^ Bonferroni adjusted), and upstream promoters (−1kb – TSS) (113% enrichment, p = 4*10^−3^ Bonferroni adjusted) (Figure 2b). In fact, our analysis revealed that methylation-sensitive Mesa elements seldom coincide with established CGI definitions (Figure 2c, Supplemental Figure 2a). Together, these findings suggest the presence of uncharacterized methylation-sensitive elements that are located beyond upstream promoter and/or promoter-CGI localizations.

Having identified hyper-sensitive MM, we next investigated their potential to directly regulate expression. We first correlated Mesa methylation with gene expression, and assessed how this compares to more commonly characterized upstream promoter regions. As MM showed most robust enrichment in gene 5’UTRs, we assessed association of methylation of Mesas in the 1 kb downstream of the TSS (thus including Mesas in 5’UTRs and those that span across both 5’UTR/exon 1) with expression, in addition to associating methylation of Mesa in upstream promoter regions. In total, we performed matched bulk RNA-seq before and after HMA treatment on 8 sample pairs for which we had corresponding WGBS methylation data.

Correlating methylation and RNA expression before and after HMA treatment in the upstream promoter (−1 kb-TSS) as compared to just Mesa sites found in the same upstream −1 kb-TSS region, identified a noticeably stronger association between Mesa methylation and gene expression (Figure 2d–e). However, the strongest association was observed when correlating the methylation of Mesas found within the 1 kb downstream of the TSS and expression. This region included 5’UTR annotated Mesas as well as those spanning the 5’UTR/exon 1 junction of genes (Figure 2f). Furthermore, our findings showed that relative methylation appears to be more akin to binary switches, rather than rheostats regarding expression control. Once the methylation drops below a particular threshold, which varies depending on the locus, expression is triggered. It is for this reason we see genes that show decreasing methylation (Figure 2e–f, horizontal axis right to left), and it is only after they approach near complete relative methylation drop (i.e., approach near 100% relative methylation drop), does expression begin to increase (Figure 2e–f, vertical axis bottom to top; Supplemental Figures 2b–h).

Thus, to summarize, by normalizing Mesa distribution percentages to genomic baselines, we demonstrated that the 5’UTR show the greatest enrichment of MM. We also showed that MM seldom overlaps with canonical CGI-defined regions. Next, by comparing the transcriptional association with regional demethylation, we observed the highest association between 5’UTR/exon 1 Mesa demethylation and gene re-activation as compared to canonical upstream promoters. Together, these findings challenge the prevailing view that promoters and promoter-CGI are the primary DNA methylation dependent regulatory regions for gene expression.

### MM are extensively marked by primed and active histone marks and highly conserved

**Figure 3.**
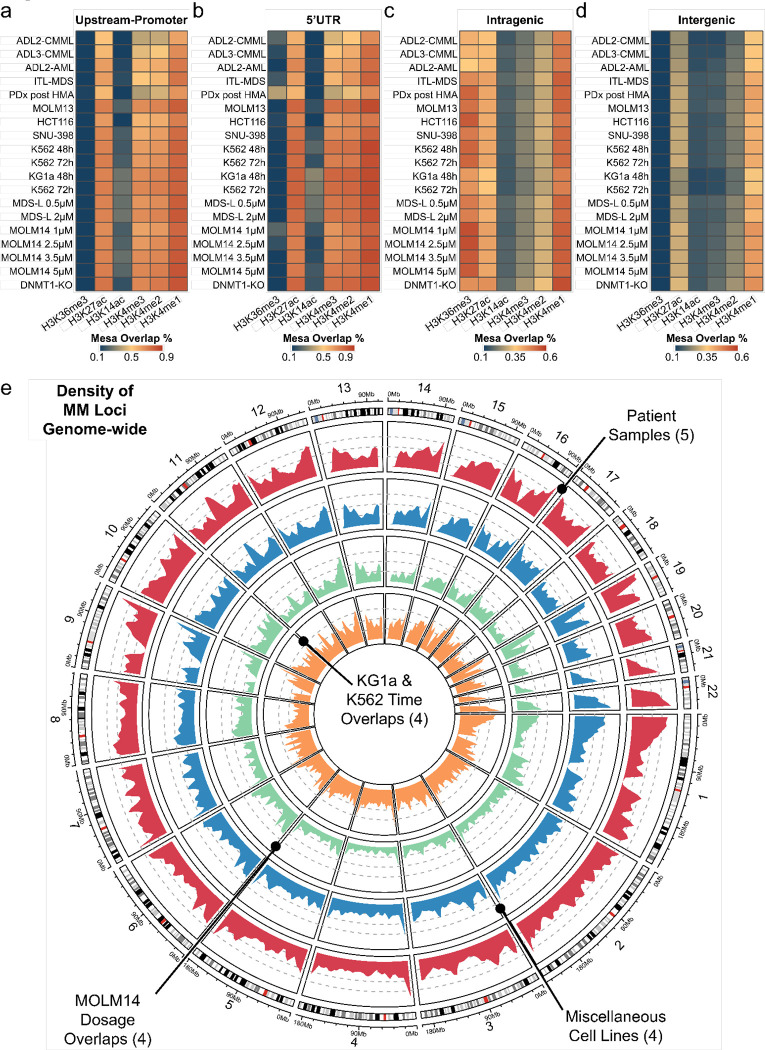
Determining the regulatory potential and location consistency of MM Predicted regulatory potential of MM is inferred by overlapping Mesa positions with a curated histone mark footprint map comprising of 60 ChIP-Seq datasets for humans, and 59 datasets for mouse. Overlap was only noted if at least 1bp overlapped between the histone peak and the Mesa with its flanking methylation buffering window (mesa width plus 300 bp in each direction). (**a**) Heatmap showing the overlap percentages between upstream-promoter Mesas (located within the region −1kb > TSS) with the specified histone mark. Heatmap showing the overlap percentages between 5’UTR Mesas with the specified histone mark. (**b**)Heatmap showing the overlap percentages between intragenic Mesas (located within exons, introns and 3’UTRs) with the specified histone mark. (**d**) Heatmap showing the overlap percentages between intragenic Mesas with the specified histone mark. (**e**) Circos plot showing the density of Mesa sites located throughout the genome for all treated samples. The red profile shows the density of Mesas as seen across all 5 profiled patient samples (ADL2-CMML, ADL3-CMML, ADL4-AML, ITL-MDS). The blue profile shows the density of Mesas as seen across 4 singular cell lines (SNU-398, HCT116, MOLM-13, HCT116 DNMT1^−/−^). The green profile shows the density of Mesa as seen across the MOLM14 samples treated with differing dosages of DAC. The orange profile shows the density of Mesa as seen across the K562 & KG1a overlapped samples which were treated with the same dosage of DAC across two differing timepoints (48 and 72hrs).

Having identified a strong association between Mesa demethylation and gene re-expression, we next questioned if Mesa overlaps with regulatory histone marks. To determine, we processed 60 active and primed histone mark datasets in human-derived lines and 59 murine-derived datasets from ENCODE and GEO to compile a histone mark footprint map that contained 6 histone marks (H3K4me1/me3, H3K14ac, H3K27ac, H3K36me3, H3K4me2 (human), and H3K4ac (mouse)) (see **Methods**). We utilized our footprint map with the strict requirement that at least 1 bp must overlap between a histone peak and an MM. Doing so revealed that promoter and 5’UTR Mesa show an (up to) 90% overlap with H3K4me1 and H3K27ac, well-characterized marks for primed and active enhancers and promoters (Figure 3a–b). Consistently, intra- and intergenic Mesa also showed strong overlap with H3K4me1 and H3K27ac marks (up to 65%), signifying a potential regulatory role of Mesas irrespective of genomic localization (Figure 3c–d), albeit likely context dependent. These findings, in conjunction with the strong association between Mesa methylation and gene expression, further supported the hypothesis that Mesas were methylation-sensitive functional regulatory elements.

If Mesas are regulatory elements, it can be assumed that their genomic positions ought to be conserved across samples. To evaluate, we overlapped Mesa genomic positions (at least 1 bp overlap) across the four patient-derived clinical samples. We observed a notable number of identical Mesas were identifiable in at least two patients (Supplemental Figure 3a). To assess how varying dosages of a HMA would affect Mesa modulation, we treated MOLM14 with four dosages of DAC (1μM, 2.5μM, 3.5μM, and 5μM; see **Methods**) and again observed overlapping percentages comparable to those seen when overlapping the patient samples (Supplemental Figure 3b). It is known that HMA treatment requires time to exert its effect. In patients, multiple rounds of HMA-therapy are required spanning months. In contrast, HMA effects can be detected in as little as 2 to 5 days in culture. Therefore, we subjected K562 and KG1a to the same AZA concentrations but different treatment durations to compare Mesa site overlap (see **Methods**). Similar to previous observations, we observed a considerable number of overlapping Mesa sites spanning at least two conditions (Supplemental Figure 3c). Collectively, these findings showed Mesa position conservation across various genetic backgrounds, drug concentrations, and treatment durations, supporting the notion that Mesa is readily modulated relatively independent of culture or treatment conditions. This observation is further supported by the consistency of Mesa site distributions observed across the human-derived samples (Figure 3e).

To conclude, we confirmed the consistently high overlap of Mesa sites with primed and active histone marks in conjunction with high numbers of conserved Mesa sites genome-wide that were conserved across different sample types, treatment dosages, treatment durations, and HMA agents. Together, these findings strongly support the adoption of Methylation Mesas as markers of precise and conserved methylation sensitive regulatory elements genome-wide. This also underscores a potential mechanistic pathway by which demethylation of a MM primes and promotes chromatin accessibility by enabling deposition of primed/activation histone marks that would culminate in sustained transcription.

### The development of CRISPR-DiR for precise demethylation to validate MM causality

In order to validate the causal regulation potential of Mesas, a fine-resolution targeted demethylation tool was required to distinguish whether gene activation is triggered by demethylation of a canonical promoter or a TSS-Mesa. Based on our analysis, canonical promoters, and TSS-Mesas can be only a few hundred base pairs apart in some gene loci, thus necessitating the use of a precise and targeted demethylation technology with higher resolution than the currently available tools (eg. CRISPR-TET1) with at least a few hundred base pairs effect window. Consequently, we aimed to engineer a demethylation tool with these more precise design requirements.

**Figure 4.**
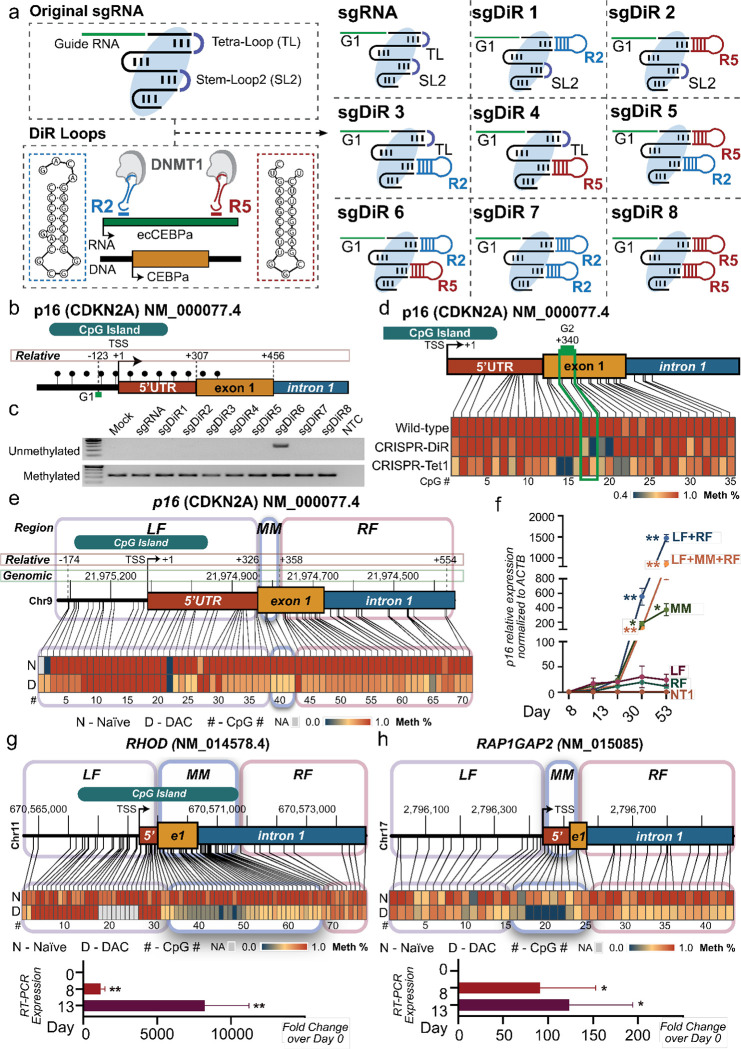
Development of CRISPR-DiR to validate causal regulatory elements for gene activation (**a**) Rationale of CRISPR-DiR design. For sgDiR, short DNMT1-interacting RNA (DiR) loops R2 and R5 from ecCEBPA^26^ were fused to the original sgRNA scaffold tetra-loop (TL) and/or stem-loop 2 (SL2) regions ^24^. The diagrams on the right represent the structure of original sgRNA control and eight different sgDiR 1–8 designs. (**b**) Schematic representation of sgRNA control and sgDiRs targeting gene *p16* proximal promoter using guide G1. Sequences for the guides, the scaffold sgRNA and sgDiRs 1–8 are listed in Supplemental Tables 3, 4. (**c**) Methylation Sensitive PCR (MSP) data demonstrating *p16* demethylation in the SNU398 line 72 h post-transfection of CRISPR systems. Mock: transfection reagents without plasmids; sgRNA: co-transfection of dCas9+sgRNA (no DiR); sgDiR1–8: co-transfection of dCas9+sgDiRs (with DiR) according to the design shown in (**a**); NTC: no template PCR control. (**d**) Methylation heatmap showing the Bisulfite Sequencing PCR (BSP) results for parental cells, CRISPR-DiR and CRISPR-TET1 stably transduced cells targeting p16 with guide G2 for 7 days. 8 colonies (representing 8 alleles) of each BSP samples were picked for sanger-sequencing, and the heatmap represents the average methylation of each CpG residue from 8 colonies for each sample. (**e**) Schematic representation of the location of LF, MM and RF in the *p16* locus. The heatmap represents the WGBS results in the LF-MM-RF region of SNU398 naïve and DAC treated cells. (**f**) Real Time-Quantitative PCR (RT-qPCR) result showing *p16* mRNA expression in SNU398 after CRISPR-DiR targeting different regions of *p16*. The CRISPR-DiR (both dCas9 and sgDiRs) are stably transduced by lentivirus. NT1: non-targeting control, LF: guides G3 and G4, MM: guides G2 and G5, RF: guides G6 and G7, LF+RF: guides G3, G4, G6 and G7, LF+MM+RF: guides G3, G4, G2, G5, G6 and G7. Significance calculated using one-tailed paired t-tests. sgDiR guides are listed in Supplemental Table 4 and the location of each region is listed in Supplemental Table 6. (**g-h**) Top: LF, MM, and RF region definition for g) RHOD and (**h**) RAP1GAP2 LF. MM is calculated by WGBS data of SNU398 naïve (**N**) and DAC (**D**) treated cells. Heatmap represent the WGBS results in the corresponding loci. Bottom: Real Time-Quantitative PCR (RT-qPCR) results showing gene mRNA expression over 2 weeks in SNU398 after CRISPR-DiR targeting LF+RF of the corresponding gene. All statistics calculated using unpaired one-tailed t-tests. All panels show mean ± SD, n = 3, *P < 0.05; **P < 0.01; ***P < 0.001.

Structural studies of the Cas9/dCas9-sgRNA-DNA complex^23^, including its derivatives (such as CRISPR-SAM^24^ and CRISPR-Rainbow^25^), revealed that the tetra-loop and stem loop 2 of the single-guide RNA (sgRNA) scaffold can be substituted with RNA aptamers, such as MS2, PP7, or boxB, without compromising stability or function. Our analysis thus far has suggested that DNMT1 likely plays a role in maintaining Mesa site methylation (Figure 1h). Previously, we have shown that DNMT1 enzymatic activity can be neutralized using DNMT1-interacting RNAs (DiRs), such as the lncRNA *ecCEBPA* identified surrounding CEBPA locus^26^. We, therefore, utilized the 23 nucleotides DiR loops, R2 and R5, from *ecCEBPA*^26^ and constructed eight modified sgDiR structures wherein the tetra- and stem-loops of the sgRNA scaffold were replaced with differing combinations of R2 and/or R5 DiRs (Figure 4a). This design diverges from conventional CRISPR-protein domain fusions, which produce bulkier complexes with wide functional effect windows, by focusing on a more streamlined engineered approach.

CDKN2A (p16^INK4a^) is one of the first characterized tumor suppressor genes whose hyper-methylated promoter is believed to be associated with gene silencing in numerous cancers^2^, including the hepatocellular carcinoma line, SNU398^27^. In order to assess the performance of candidate CRISPR-DiR designs while excluding guide efficiency concerns, we started with guide G1, which was previously used^24^ to target 123 bp upstream of p16 TSS (Figure 4b). Guided by G1, dCas9 was co-transfected with either unmodified sgRNA or modified sgDiR1–8 into SNU398. Seventy-two hours post-transfection, the p16 locus demethylation was utilized as the experimental readout, revealing sgDiR6 as the optimal structural to trigger targeted demethylation (Figure 4c). This became the finalized CRISPR-DiR design henceforth.

To compare CRISPR-DiR demethylation efficiency and accuracy with existing tools, we performed a head-to-head comparison between CRISPR-DiR and CRISPR-TET1 (Figure 4d). For this, we used a second guide, G2, targeting p16 exon 1 to directly assess the efficacy and precision of both technologies using the same guide target. The use of G2 also provided additional confirmation for the reproducibility of the CRISPR-DiR function in addition to our prior testing with G1. Utilizing bisulfite sequencing PCR and Sanger sequencing, we assessed the single-base resolution demethylation profiles of both technologies. In our testing, CRISPR-TET1 showed an effect window of at least 350 bp surrounding the targeted locus, a finding that is comparable to previous studies. In contrast, CRISPR-DiR was capable of introducing demethylation with an effect window size of approximately 30 bp, meaning it had an effect window that is over 10x more precise than other tested technologies (Figure 4d). This superior effect window accuracy of CRISPR-DiR, therefore, made it ideal for validating Mesa demethylation causal effects.

Although CRISPR-DiR introduced demethylation at *p16* when guided by G1 (Supplemental Figure 4a), this proximal promoter target, which is the well-believed optimal target region, resulted in only a moderate increase in *p16* mRNA (Supplemental Figure 4b), suggesting that *p16* proximal promoter demethylation might not strongly correlate with gene expression. Furthermore, the *p16* promoter-CGI (Figure 4e – CpG residues 0–20), the target of guide G1, did not show noticeable demethylation changes post-DAC treatment in the SNU398 WGBS (Figure 4e). Fortuitously, our analysis identified an MM downstream of the *p16* TSS at the 5’UTR/exon 1 junction (Figure 4e). This made *p16* an ideal candidate to compare the gene re-activation efficiency of Mesa- targeted demethylation as compared to commonly investigated upstream promoter-CGI-targeted demethylation.

Based on the Mesa profiles observed across all samples, we observed a consistent methylation buffering window that flanked Mesas at a distance equivalent to ~3-nucleosomes away (~441 bp; Figure 1e–g, Supplemental Figure 1). In SNU398 cells, the p16 TSS-Mesa was found to be located between +326 and +358 bp relative to the TSS (at the p16 5’UTR/exon 1 junction). In comparison, the proximal promoter targeted by guide G1 was located at −123 bp relative to the TSS, meaning it was situated within the left flank (LF) buffering window. We, therefore, designed two guides in each window targeting the *p16* MM, LF, and the right flank (RF) buffering window. Using CRISPR-DiR, we precisely demethylated each targeted region without affecting nearby untargeted regions (Supplemental Figure 4c), enabling causal assessment of locus demethylation and subsequent gene re-expression (Figure 4f). Intriguingly, when targeting LF, MM, and RF independently, sole demethylation of MM resulted in significantly higher p16 re-expression than individual targeting of either LF or RF (Figure 4f). Notably, consistent with previous promoter-CGI targeting using G1, only moderate gene activation was observed when targeting the LF situated in the upstream promoter region, even when utilizing two extra guides via a lentiviral stable system for longer treatment (Figure 4f). Consistent with the analysis that TSS-Mesas are more enriched in 5’UTR regions, these validations at the p16 locus support the greater observed association between Mesa demethylation and transcription.

Having successfully induced gene re-expression using single region targeting, we next tested whether combinatorial region targeting could induce expression more potently. To that end, we observed that targeting both Mesa boundaries simultaneously, LF+MM+RF or LF+RF, was capable of inducing even greater *p16* mRNA expression than targeting singular region targeting (Figure 4f). Our observations, therefore, led us to hypothesize that Mesas, with their flanking buffering regions, may signify core methylation-sensitive regulatory elements, as targeting the flanking boundaries of Mesas appeared to be the most effective method for complete demethylation and maximal gene activation (Figure 4f). As a validation of our findings, we observed comparable p16 activation utilizing the same targeting strategy in the U2OS human osteosarcoma cell line, which also has hypermethylated and silenced *p16* (Supplemental Figures 4d, 4e). To ensure CRISPR-DiR specificity, we tested and observed no off-target activation from the adjacent hypermethylated and silenced *p14* gene*,* or the distant *CEBPA* gene from which we adopted the DiR loops (located on another chromosome and actively expressed) (Supplemental Figure 4f).

In order to test our Mesa targeting strategy, we tested Mesa-induced gene activation of three additional methylated and silenced tumor suppressor gene loci in SNU398 cells. These included *RHOD* (Figure 4g) and *CDX1* (Supplemental Figure 4g), both of which have Mesa sites that do not overlap with upstream TSS promoters. Additionally, we tested Mesa-induced gene activation for the tumor suppressor gene *RAP1GAP2* (Figure 4g) which, in contrast to the previously tested genes, has *no* annotated promoter-CGI. These results confirm the effectiveness of our targeting strategy in reactivating methylated and silenced genes, regardless of annotated CGI presence.

Our data, therefore, demonstrates that CRISPR DiR offers a demethylation approach with higher resolution than current TET1-based methods. Additionally, our findings suggest that direct demethylation of MM, or their adjacent flanking regions, leads to more robust gene activation than traditional upstream TSS-proximal promoter target regions.

### Tracing the epigenetic kinetics triggered by p16 MM demethylation

**Figure 5.**
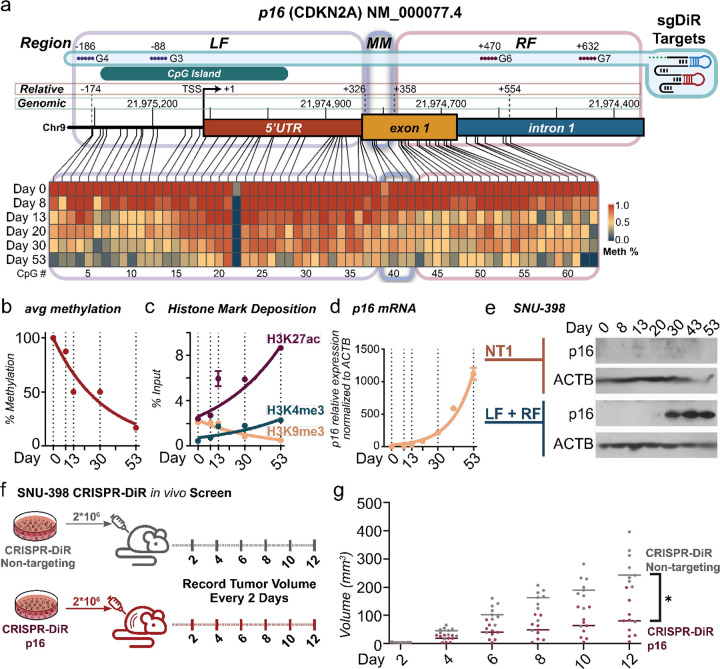
Kinetics tracing of MM induced demethylation of p16 (**a**) Schematic representation of CRISPR-DiR targeting *p16* LF+RF. BSP methylation map indicating the gradual demethylation profile starting from the targeted LF and RF locus and gradual spreads inwards towards the Mesa locus. The methylation map for each time point is generated using at least 8 BSP colonies. (**b**) The kinetics of average methylation changes of CRISPR-DiR LF+RF targeted samples as indicated in (**a**). (**c**) ChIP-qPCR results showing the gradual increase of active histone markers (H3K4Me3, H3K27Ac) and decrease of silencing marker (H3K9Me3) enrichment in the *p16* CRISPR-DiR LF+RF targeted cells. (**d**) Real Time-Quantitative PCR (RT-qPCR) result showing the gradual increase of *p16* mRNA expression after CRISPR-DiR targeting *p16* LF+RF in SNU398 cells. (**e**) Western Blot assessing p16 protein after CRISPR-DiR treatment. β-actin (ACTB) was used as internal control. (**f, g**) Tumor growth *in vivo* following xenograft nine mice of CRISPR-DiR non-targeting control (NT1) or p16 (LF+RF) targeted SNU398 cells. Statistical significance between tumour sizes in (**i**) was calculated using a two-tailed paired t-test. All panels show mean ± SD, n = 3, *P < 0.05; **P < 0.01; ***P < 0.001.

As CRISPR-DiR triggers targeted demethylation in a manner that is more native to the cell without necessitating the introduction and recruitment of foreign cellular machinery, we observed that a progressive increase of *p16* mRNA transcription takes weeks to occur following targeted demethylation (Figure 4f). This prompted us to trace the kinetics of epigenetic changes and *p16* expression following MM demethylation for an equivalent time frame. To that end, single CpG site methylation (Figure 5a), locus average methylation (Figure 5b), histone mark deposition (Figure 5c), mRNA (Figure 5d), and protein levels (Figure 5e) were tracked for 53 days following CRISPR-DiR targeting *p16* Mesa LF+RF in SNU398 cells.

As we tracked the kinetics changes through our time points, we began to see LF and RF demethylation at our guide target sites from around Day 8, with demethylation spreading towards the interjacent Mesa site from Day 13 onwards (Figure 5a). This spreading of methylation was observed to travel inwards towards the Mesa, but never spread beyond the LF and RF boundaries to upstream/downstream flanking regions (Upstream left flank (ULF), downstream right flank (DRF)) for the entire 53-day tracking period (Supplemental Figure 5a, b). Thus, we can conclude that methylation changes remain confined to the Mesa and its immediate boundaries, enabling us to infer that Mesas and their methylation buffering windows function as core methylation-sensitive regulatory elements in our test cases. These findings also potentially explain why CRISPR-DiR boundary targeting represents an optimal strategy for gene activation.

As we followed our kinetics profiling further, we observed a gradual decrease of average methylation across the LF-MM-RF locus over the 53 days (Figure 5b). Additionally, post-Day 8 demethylation, we observed increases in the deposition of active histone marks (H3K4me3, H3K27ac) and a decrease of the silencing mark H3K9me3 between Days 8–13 (Figure 5c). Over the same time period, we also observed *p16* mRNA expression increased significantly after Day 13 (Figure 4f, 5d), with visible p16 protein levels detected after Day 20 (Figure 5e). This indicated that demethylation preceded transcriptional activation and protein expression, triggering histone modifications to accommodate subsequent gene expression. Together, these findings show the native cellular progression of demethylation, followed by transcription re-activation.

To evaluate whether demethylation, once initiated, had a lasting effect, we generated an inducible CRISPR-DiR SNU398 cell line, wherein dCas9 can be conditionally induced and expressed with doxycycline. Following three days of induction, we observed *p16* demethylation and activation that was maintainable for over a month (Supplemental Figure 5c, d). These findings therefore suggest that Mesa are epigenetic regulatory elements, demethylation of which, induces self-sustaining and long-term histone modifications and chromatin rewiring events that can sustain active gene expression.

Finally, to assess our Mesa targeting strategy and the efficacy of CRISPR-DiR *in vivo*, we performed xenograft studies in mice. Using the SNU398 line, CRISPR-DiR non-targeting control and *p16* targeted cells were injected into mice, and tumor size was traced for 12 days (Figure 5f). We observed that *p16* targeted mice tumors were significantly smaller than controls (Figure 5g), demonstrating the therapeutic potential of a targeted epigenetic therapy based on precise Mesa targeting with CRISPR DiR.

### Mesa demethylation and gene re-expression initiate long-range interaction with distal Mesa loci

Thus far, we have shown that targeted demethylation of the p16 Mesa was sufficient to trigger potent gene expression. Additionally, our inducible CRISPR-DiR findings (Supplemental Figure 5c, d) suggested that subsequent chromatin rewiring events might occur, resulting in lasting effects long after cessation of CRISPR-DiR induction.

**Figure 6.**
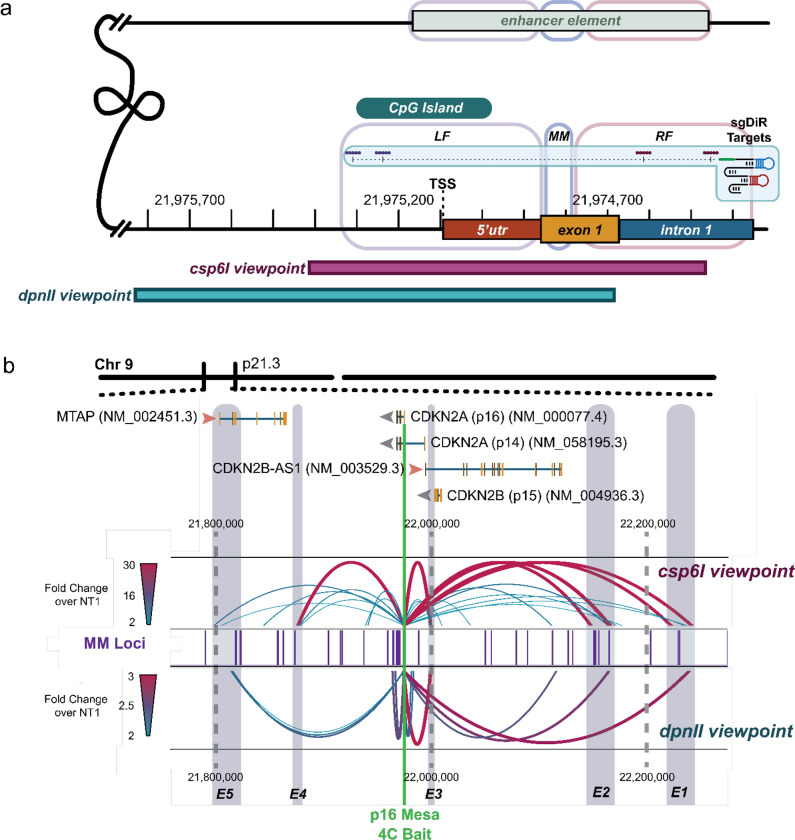
CRISPR-DiR induced local demethylation in *p16* initiates strong interactions with distal elements marked as MM (**a**) Schematic representation of the two viewpoints (generated by restriction enzyme Csp6I and DpnII) surrounding the 800 bp demethylated p16 LF-MM-RF region used for Circularized chromosome conformation capture (4C)-Seq. (**b**) 4C-Seq demonstrating the distal interactions initiated by CRISPR-DiR targeted p16 demethylation in SNU398 cells. The interaction changes of p16 LF-MM-RF demethylated sample was normalized to the same time point (Day 13) non-targeting control (NT1) sample. The top panel shows distal interactions captured for Viewpoint Csp6I while the bottom shows Viewpoint DpnII. The MM annotations are indicated with purple bars in the middle. The strong interaction changes demonstrated by the interaction arcs, with color from blue to red, representing the interaction fold change from two-fold to the highest fold change. The potential distal enhancer elements with the strongest interaction were highlighted for both viewpoints on the top, labeled from *p16* upstream to downstream (negative orientation, right to left) as E1, E2, E3, E4, E5, and E6.

Previous studies have reported a *p16* enhancer located ~150 kb upstream of the *p16* TSS^28–30^. Nonetheless, long-range interactions with the *p16* locus, and the impact of locus-specific demethylation on enhancer interactions, remain unexplored. To assess the impact of LF-MM-RF demethylation on the 3D topology surrounding the *p16* locus, we performed Circularized Chromosome Conformation Capture (4C) for CRISPR-DiR non-targeting or *p16* LF+RF targeted samples. We designed two viewpoints (“baits”) close to the LF-MM-RF locus (Figure 6a). Comparing the targeted *p16* demethylated region (LF+MM+RF total ~800bp) with the non-targeting (NT1) control, we detected noticeable interaction changes between distal elements and the *p16* demethylated locus, several of which were consistent across both viewpoints (Figure 6b). Consistent with the previously reported *p16* enhancer region^28–30^, we observed that this distal locus (E2) interacted with the demethylated *p16* Mesa (Figure 6b). Additionally, we detected novel interactions at sites >200 kb upstream of *p16* (E1), at sites within the *Anril*-*p15-p14* locus (E3), and at sites >100 kb downstream of *p16* (E4, E5). The strongest interactions were validated by their overlap between the two viewpoints, including the known E2 enhancer. Furthermore, by overlapping SNU398 Mesas with the 4C interaction landscape, we observed that many distal interacting loci initiated by *p16* promoter Mesa demethylation aligned closely with intragenic and intergenic Mesas (Figure 6b), indicating the Mesa signature demarcates both proximal and distal functional regulatory elements.

Together, through targeted Mesa demethylation via CRISPR-DiR followed by 4C, we successfully captured the previously reported p16 enhancer region while also identifying potentially novel *p16* enhancer elements, including a close, previously unreported interplay between *p16* and the *Anril-p15-p14* gene locus. Intriguingly, both promoter and distal interacting loci were found to be highly concordant with Mesa localizations. These findings, therefore, strengthen the notion that MM are self-sustaining epigenetic regulatory elements that maintain long-term gene activation through focused demethylation only within the Mesa core that results in subsequent histone modifications and chromatin rewiring events that interact with distal elements also marked as Mesas.

## Discussion

Over the years, numerous publications have searched for functional methylation-sensitive regulatory elements. This has led to the identification of numerous DNA methylation landscape elements such as CpG shores^15^, shelves^31^, canyons^32^, and valleys^33^. However, definitive data on which elements’ demethylation triggers gene re-expression remains scarce. It is possible that this could have been due to technological limitations or a lack of a precise definition of what constitutes a functional seed methylation regulatory element and where they are located genome-wide.

In this study, we report a previously uncharacterized genome-wide causal methylation-sensitive regulatory element, **M**ethylation **M**esas (MM). MM are narrow-width methylation-sensitive seed loci, predominantly between 45–294 bp in width. The observed MM signature was consistent across all human and mouse cell lines and patient-derived samples profiled in this study. Genome-wide, Mesas were more enriched in gene 5’UTRs (downstream-TSS) (~155% enrichment over baseline) than more commonly investigated upstream-TSS regions (−1 kb > TSS) (~113% enrichment over baseline). Coupling whole-genomic bisulfite sequencing (WGBS) with matched bulk RNA-seq data, we observed a substantially greater association between the demethylation of 5’UTR/exon 1 Mesas and transcriptional activation than upstream-TSS promoter demethylation.

To assess Mesa functionality, we engineered and developed CRISPR-DiR, a precisely targeted demethylation technology with functional characteristics that enabled accurate elucidation of Mesa causality. Specifically, using the important tumor suppressor p16 as a model, we validated that more robust gene activation can be achieved by targeted demethylation of the 5’UTR/exon 1 Mesa rather than the upstream promoter-CGI. This finding is consistent with other published findings from our lab^34,35^ in which Mesa targeting was sufficient to induce strong gene re-expression. Collectively, both genome-wide analyses, in conjunction with individual validations, support the notion of MM as causal methylation-sensitive regulatory elements.

While validating the p16 locus, we observed that simultaneous targeting of Mesa LF and RFs represented the simplest and most effective strategy tested for triggering demethylation and inducing gene expression. Intriguingly, although CRISPR-DiR only introduced demethylation in the guide targeted regions at an early time point, we observed that the triggered demethylation wave at the flanking boundaries initiates a stepwise process whereby methylation spreads inwards to the Mesa, but not outside of the LF-MM-RF locus, even after 53 days of kinetics tracing. This observation suggests a model where Mesas, together with their bi-directional methylation buffering boundaries, are core demethylation cassettes that are self-contained through an as yet unknown mechanism. Additional studies are required to further investigate the mechanism shaping Mesas, their boundaries, and how cells recognize these functional core elements be it through conserved sequences or other means.

A related aspect of this study was the long-term kinetics tracing following p16 TSS-proximal Mesa demethylation. After CRISPR-DiR introduced Mesa cassette demethylation, we observed the acquisition of active histone marks, and removal of repressive marks, followed by gradual gene re-expression and increased protein production over a period of nearly two months. Our inducible CRISPR-DiR assay also illustrated that in as short as three days, Mesa demethylation treatment could enable over a month of maintenance of the demethylation status and gene activation. Together, these dynamic processes indicate a model where Mesa demethylation could further trigger local chromatin modifications to gradually enable and maintain an active transcription environment.

When annotating Mesas with our complied histone footprint map, we observed high concordance between Mesas and H3K4me1 across all genomic compartments. H3K4me1 was initially believed to pre-mark primed enhancers and fine-tune their activity with the further acquisition of H3K27ac^36,37^. Later, it was also reported to mark both active and bivalent promoters^38^. Additionally, some researchers propose that enhancer and promoter elements share similar structural (histone) features and should be considered as a single regulatory element with varied levels of RNA transcription^39^. The priming feature of H3K4me1 has also been reported to represent a ‘window of opportunity’, where regulatory activity is waiting to occur once triggered by activation signals^40^. Therefore, the high concordance of priming H3K4me1 mark with Mesa locations genome-wide further supports the notion that Mesas are functional regulatory elements.

In this study, we validated Mesa function in TSS-proximal regions because they are the most well-understood regulatory region throughout the genome with a cis gene expression readout. However, when capturing distal looping interactions initiated by TSS-proximal Mesa demethylation, we observed that many distal elements that interacted with our targeted TSS-proximal Mesa were also annotated as (distal-) Mesa sites. It was striking that the demethylation of a small Mesa locus (~800 bp) was capable of inducing chromatin reorganization as far away as 500 kb, verifying that localized, specific modulation of DNA methylation was capable of broadly impacting chromatin configuration and was capable of controlling gene expression along with distal regulatory elements. The distal-Mesas associated with the induced looping interactions localize in both intragenic and intergenic compartments, many of which were marked by H3K4me1 and H3K27ac, indicating Mesa not only demarcate regulatory function proximal to TSS, but also mark intragenic and intergenic regulatory elements such as enhancers. It therefore appears that although Mesas can be identified through a singular definition, the function of a Mesa appears to be context-specific, be it a proximal-TSS expression regulatory element, or an enhancer element located distal to a target gene. It would be insightful to conduct further studies to better understand the relationship between/of distal-Mesas and enhancers, as well as the recognition mechanism between TSS-proximal and distal-Mesas.

Additionally, in spite of many years of study, it is still not clear how mutations in DNA methyltransferase 3A (DNMT3A) can lead to clonal hematopoiesis of indeterminate potential (CHIP)^41^, myelodysplastic syndrome (MDS)^42,43^ and Acute Myeloid Leukemia (AML)^44,45^. More so, despite their use for decades, it is still not entirely clear how global hypomethylating agents such as 5-azacytidine and decitabine induce responses in MDS and AML. In light of this study though, it is plausible that “we have been looking in the wrong places”, and future studies are required to investigate Mesa demethylation in these disease contexts and how their modulation tracks with genomic and regulatory events, and additionally, whether Mesas can be utilized for clinical prognostication.

In conclusion, our study presents novel insights into the locations of functional methylation-sensitive regulatory loci in humans and mice, and provides a precise new technology to regulate them in an accurate and targeted manner. In addition, our *in vivo* findings highlight the efficacy and translatable potential of utilizing MM targeting in conjunction with CRISPR-DiR to activate tumor suppressors. Purely based on the fusion of RNA stem loops, the delivery size of CRISPR-DiR is substantially smaller than most other CRISPR-based technologies tethering functional protein domains, which would better accommodate the size limitations of delivery cargo and potentially minimize toxicities. The combination of precise Mesa-defined regulatory elements targeted by CRISPR-DiR can enable the development of more precise functional biomarkers or targeted epigenetic-based therapies.

## Materials and Methods

### Cell-lines and Patient Samples

The cell lines SNU398, K562, KG1a, MOLM13, MOLM14, MDS-L were cultured in Roswell Park Memorial Institute 1640 medium (RPMI) (Life Technologies, Carlsbad, CA) supplemented with 10% fetal bovine serum (FBS) (Invitrogen). Human HEK293T and human osteosarcoma cell line U2OS were maintained in Dulbecco’s Modified Eagle Medium (DMEM) supplemented with 10% fetal bovine serum (FBS). HCT116 was cultured in McCoy’s 5a Medium (Catalog number:16600082). All cell lines were maintained at 37°C in a humidified atmosphere with 5% CO2 and cultured in the absence of antibiotics if not otherwise specified. The ADL samples were mononuclear cells from patients with acute myeloid leukemia and chronic myelomonocytic leukemia. They were obtained from the Myelodysplastic Registry and South Australian Cancer Research Biobank (Institutional Central Adelaide Local Health Network Ethics approval No. R20110304) housed at the South Australian Health and Medical Research Institute. Matched DNA and RNA samples were obtained from the same patient at diagnosis and after four cycles of Azacytidine treatment. The ITL myelodysplastic syndrome sample was obtained from the University of Eastern Piedmont, Novara, Italy (Protocol 939/CE, Study No. CE 154/15). Matched DNA and RNA samples were obtained from the same patient at diagnosis and after one cycle of Azacytidine treatment. Samples were handled in accordance with the Declaration of Helsinki. The CT-26 and PDx data was obtained from GEO Series GSE192745. Additional HCT116 data was obtained from GEO Series GSE97889.

### RNA isolation

Total RNA was either extracted using AllPrep DNA/RNA Mini Kit (Qiagen, Valencia, CA) and treated with RNase-free DNase Set (Qiagen) following the manufacturer’s instructions, or isolated with TRIzol (Invitrogen). If the RNA isolation was carried out with the TRIzol method, RNA samples were treated with recombinant RNase-free DNase I (Roche) (10 U of DNase I per 3 mg of total RNA; 37°C for one hour; in the presence of RNase inhibitor). After DNase I treatment, RNA samples were extracted with acidic phenol (pH 4.3) to eliminate any remaining traces of DNA.

### Genomic DNA extraction

Genomic DNA was extracted by either the AllPrep DNA/RNA Mini Kit (Qiagen, Valencia, CA) for BSP, MSP, and COBRA assays or by Phenol–chloroform method if extremely high- quality DNA samples were required for whole genomic bisulfite sequencing (WGBS). The Phenol–chloroform DNA extraction was performed as described^46^*.* In the case of the SNU398 samples, 10 cm plates of wild type SNU398 cells and Decitabine treated SNU398 cells were washed twice with cold PBS. 2 mL of gDNA lysis buffer (50mM Tris-HCl pH 8, 100mM NaCl, 25mM EDTA, and 1% SDS) was added directly to the cells. The lysates were incubated at 65°C overnight with 2mg of proteinase K (Ambion). The lysate was diluted 2 times with TE buffer before adding 1mg of RNase A (PureLink) and followed by a one-hour incubation at 37°C. The NaCl concentration was subsequently adjusted to 200mM followed by phenol-chloroform extraction at pH 8 and ethanol precipitation. The gDNA pellet was dissolved in 1mL TE pH 8 buffer and incubated with RNase A with a concentration of 100ug/mL (Qiagen) for 1 hour at 37°C. The pure gDNA was recovered by phenol-chloroform pH 8 extraction and ethanol precipitation and dissolved in TE pH 8 buffer.

### 5-aza-2’-deoxycytidine (Decitabine) treatment

SNU398 cells were treated with 2.5 μM of 5-aza-2’-deoxycytidine (Sigma-Aldrich) according to the manufacturer’s instructions. MOLM13 cells were treated with 1 μM of 5-aza-2’-deoxycytidine. MOLM14 cells were treated with either 1 μM, 2.5 μM, 3.5 μM, or 5 μM 5-aza-2’-deoxycytidine. MDS-L cells were treated with either 0.5 μM or 2 μM 5-aza-2’-deoxycytidine. Media and drug were refreshed every 24 h. RNA and genomic DNA were isolated after 3 Days (72 h) treatment. Details on how the HCT116 sample was treated can be found in the respective publication^47^.

### 5-azacytidine (Azacytidine) treatment

K562 and KG1a cells were treated with 2 μM of 5-azacytidine (Sigma-Aldrich) according to the manufacturer’s instructions. Cells were treated for either 48 or 72 hours with media and rug refreshed every 24 h. RNA and genomic DNA were isolated thereafter.

### Deoxycytidine (Dox) treatment

In the dCas9 Tet-On SNU398 cells (inducible CRISPR-DiR system shown in Supplemental Figure 5c–d), the same targeting strategy shown in Figure 5a (LF + RF) was used, and dCas9 expression was induced following treatment with Deoxycytidine (Dox). Dox was freshly added to the culture medium (1 μM) every day for Dox+ sample, while Dox- samples were cultured in normal medium without Dox. For Dox induced 3 days/8 days samples, 1 μM Dox was added to fresh medium for 3 days and 8 days accordingly, then the cells were kept in medium without Dox until Day 32; for Dox induced 32 days samples, 1 μM Dox was added to fresh medium every day for 32 days. All treated cells were c*ul*tured and assayed at Day 3, Day 8 and Day 32.

### Transient transfections

SNU398 cells were seeded at a density of 3.5 × 10^5^ cells/well in 6-well plates 24 hours before transfection employing jetPRIME transfection reagent (Polyplus Transfection) as described by the manufacturer. 2 μg mix of sgRNA/sgDiR and dCas9 plasmid(s) (sgRNA/sgDiR: dCas9 molar ratio 1:1) were transfected into each well of cells. The culture medium was changed 12 hours after transfection. Alternatively, the Neon^™^ Transfection System (Thermo Fisher) was used for cell electroporation according to the manufacturer’s instructions. The same plasmid amount and ratios were used in the Neon as in the jetPRIME transfection. The parameters we used for the highest SNU-398 transfection efficiency were 0.7 to 1.5 million cells in 100 μl reagent, voltage 1550 V, width 35 ms and 1 pulse. The culture medium was changed 24 hours after transfection. The whole scaffold sequences of sgRNA and sgDiR structures are listed in **Supplemental Table 3.** The guide RNA sequences are listed in **Supplemental Table 4.** The plasmids used in this study are listed in **Supplemental Table 5**.

### Lentivirus production

pMD2.G, psPAX2, and lentivector (plv-dCas9-mCherry, pcw-dCas9-puro, plv-NT1-sgDiR-EGFP, plv-G3sgDiR-EGFP, plv-G4sgDiR-EGFP, plv-G2sgDiR-EGFP, plv-G5sgDiR-EGFP, plv-G6sgDiR-EGFP, plv-G7sgDiR-EGFP, and all the rest of sgDiR constructs with different gRNA sequences list in **Supplemental Table 4**) were transfected into 10 million 293T using TransIT-*LT1* reagent (Mirus), lentivector: psPAX2: pMD2.G 9 μg: 9 μg:1 μg. The medium was changed 18 hours post-transfection, and the virus supernatants were harvested at 48hr and 72hr after transfection. The collected virus was filtered through 0.45 μm microfilters and stored at −80 °C. The plasmids used in this study are listed in **Supplemental Table 5**. To note, few studies have tried to modify the sgRNA scaffold to increase their stability. One option is to remove the putative POL-III terminator (4 consecutive Ts in the beginning of sgRNA scaffold) by replacing the fourth T to G ^48^. Thus, in the CRISPR-DiR design, the fourth T (in bold, italic, underline below) was substituted with G, to make the structure more stable by enabling efficient transcription, while keeping substantially the same secondary structure and decreasing the minimum free energy (MFE). Accordingly, the corresponding A was substituted with C to preserve base-pairing with the “G”. All the sgRNA, sgDiRs scaffold sequences are listed in **Supplemental Table 3**, guide RNA sequences are listed in **Supplemental Table 4** and the locations of each region (Region ULF, LF, MM, RF, and DRF) are listed in **Supplemental Table 6**.

### Generating CRISPR-DiR stable and inducible cell lines

Both SNU398 and U2OS cells were seeded in T75 flasks or 10cm plates 24 h prior to transduction, and were first transduced with dCas9 or inducible dCas9 virus medium (thawed from −80 °C) together with 4 μg/mL polybrene (Santa Cruz) to make SNU-398-dCas9, U2OS-dCas9, or inducible SNU-398- dCas9 stable lines. Once incubated for 24 hours at 37°C in a humidified atmosphere of 5% CO2, the medium with virus can be changed to normal culture medium. The dCas9 positive cells were sorted using a mCherry filter setting with a FACS Aria machine (BD Biosciences) at the Cancer Science Institute of Singapore flow cytometry facility, while the inducible dCas9 positive cells were selected by adding puromycin at 2 μg/ml concentration in the culture medium every other day. The cells were further cultured for more than a week to obtain stable cell lines. Once the dCas9 and inducible dCas9 cell lines were generated, sgDiRs virus with different guide RNAs were mixed in equal volume and transduced into dCas9 or inducible dCas9 stable lines with the same method as described above. The sgDiRs used for generating each stable cell line, the location of each sgDiR, can be found in **Supplemental Table 4.** The definition of LF, MM and RF can be found in **Supplemental Table 6**. All sgDiR stable cell lines were sorted using an EGFP filter with a FACS Aria machine (BD Biosciences) at the Cancer Science Institute of Singapore flow cytometry facility, and further assessed in culture by checking the efficiency by microscopy regularly.

### Quantitative Real-time PCR (qRT-PCR)

1 ug of RNA was reverse transcribed using Qscript cDNA Supermix (QuantaBio). The cDNAs were diluted 3 times for expression analysis. qRT-PCR on cDNA or ChIP-DNA were performed on 384 well plates on a QS5 system (Thermo Scientific) with GoTaq qPCR Master Mix (Promega, Madison, WI). The fold change or percentage input of the samples was calculated using the QuantStudio^™^ Design & Analysis Software Version 1.2 (ThermoFisher Scientific) and represented as relative expression (ΔΔCt). All measurements were performed in triplicate. Primers used in this study are listed in **Supplemental Table 7**.

### Western Blot Analysis

Total cell lysates were harvested in RIPA buffer (150mM NaCl, 1% Nonidet P-40, 50mM Tris, pH8.0, protease inhibitor cocktail) and protein concentrations were determined by Bradford protein assay (Bio-Rad Laboratories, Inc. Hercules, CA, USA) and absorbance was measured at 595 nm on the Tecan Infinite^®^ 2000 PRO plate reader (Tecan, Seestrasse, Switzerland). Equal amounts of proteins from each lysate were mixed with 3X loading dye and heated at 95 °C for 10 minutes. The samples were resolved by 12% SDS-PAGE (running buffer: 25 mM Tris, 192 mM Glycine, and 0.1% SDS) and then transferred to PVDF membranes (transfer buffer: 25 mM Tris, 192 mM Glycine, and 20% (v/v) methanol (Fischer Chemical)). Membranes were blocked with TBST buffer containing 5% skim milk one hour at room temperature with gentle shaking. The blocked membranes were further washed three times with TBST buffer and incubated at 4°C overnight with primary antibodies CDKN2A/p16INK4a (ab108349, Abcam, 1:1000), β-actin (Santa Cruz β-actin (C4) Mouse monoclonal IgG1 #sc-47778, 1:5000), followed by HRP-conjugated secondary antibody incubation at room temperature for one hour. Both the primary and secondary antibodies were diluted in 5% BSA-TBST buffer, and all the incubations were performed in a gentle shaking manner. The immune-reactive proteins were detected using the Luminata Crescendo Western HRP substrate (Millipore).

### Bisulfite treatment

The methylation profiles were assessed by bisulfite-conversion based assays. For DNA bisulfite conversion, 1.6–1.8 μg of genomic DNA of each sample was converted by the EpiTect Bisulfite Kit (Qiagen) following the manufacturer’s instructions.

### Methylation-Specific PCR (MSP), Combined Bisulfite Restriction Analysis (COBRA) and bisulfite sequencing PCR (BSP)

The bisulfite converted DNA samples were further analyzed by three different PCR based methods in different assays for the methylation profiles. For Methylation-Specific PCR (MSP), both methylation specific primers and unmethylation specific primers of *p16* were used for the PCR of the same bisulfite converted sample (the transient transfection samples in the sgRNA and sgDiR1–8 screening assay). The PCR was performed with ZymoTaq PreMix (ZYMO RESEARCH) according to the manufacturer’s instructions, with the program: 95°C 10min, 35 cycles (95°C 30s, 56°C 30s, 72°C 1min), 72°C 7min, 4°C hold. Two PCR products of each sample (Methylated and Unmethylated) were obtained and analyzed in 1.5% agarose gels.

For Combined Bisulfite Restriction Analysis (COBRA), primers specifically amplify both the methylated and unmethylated DNA (primers annealing to specific locus without any CG site) in each region were used for the PCR of the bisulfite converted samples. The PCR was performed with ZymoTaq PreMix (ZYMO RESEARCH) according to the manufacturer’s instructions, with the program: 2 cycles (95°C 10min,55°C 2min, 72°C 2min), 38 cycles (95°C 30s, 55°C 2min, 72°C 2min), 72°C 7min, 4°C hold. The PCR products were therefore loaded in a 1% agarose gel and the bands with predicted amplification size were cut out and gel purified. 400ng purified PCR fragments were incubated in a 20 μl volume for 2.5h-3h with 1 μl of the restriction enzymes summarized in **Supplemental Table 8**. 100ng of the same PCR fragments were incubated with only the restriction enzyme buffers under the same conditions as uncut control. The uncut and cut DNA were then separated on a 2.5% agarose gel and stained with ethidium the bromide.

For bisulfite sequencing PCR (BSP), primers specifically amplify both the methylated and unmethylated DNA (primers annealing to the specific locus without any CG site) were used for the PCR of the bisulfite converted samples. The PCR was performed with ZymoTaq PreMix (ZYMO RESEARCH) according to the manufacturer’s instructions, with the program: 2 cycles (95°C 10min,55°C 2min, 72°C 2min), 38 cycles (95°C 30s, 55°C 2min, 72°C 2min), 72°C 7min, 4°C hold. PCR products were gel-purified (Qiagen) from the 1% TAE agarose gel and cloned into the pGEM-T Easy Vector System (Promega) for transformation. The cloned vectors were transformed into Stbl3 competent cells and miniprep was performed to extract plasmids for Sanger sequencing with either sequencing primer T7 or SP6. Sequencing results were analyzed using QUMA (Quantification tool for Methylation Analysis). Samples with conversion rate less than 95% and sequence identity less than 90% as well as clonal variants were excluded from our analysis. The minimum number of clones for each sequenced condition was 8. To note, p16 gene in HCT116 cell line has one mutated and unmethylated allele, and another wild-type and hypermethylated allele. Therefore, for BSP assay in HCT116, The mutated and unmethylated p16 allele were excluded from the analysis.

All the MSP, COBRA, and BSP primers as well as restriction enzymes can be found in **Supplemental Table 8**.

### Whole Genomic Bisulfite Sequencing (WGBS)

For samples handled in-house, WGBS was performed by BGI (Beijing Genomics Institute). BGI performed library construction and sequencing. Samples were sequenced with the aim of achieving approximately 30X human genome coverage with 2X150 paired end reads.

### Xenograft murine model

All experiments in mice were conducted according to our protocols approved by the Institutional Animal Care and Use Committee under National University of Singapore. Mice of similar ages were tagged and grouped randomly for control and test treatments. 2.5 million SNU-398 HCC cells were injected into the flanks of NOD-SCID mice after transduction with 1) CRISPR-DiR non-targeting control GN2, or 2) CRISPR-DiR targeting *p16* region LF+RF*.* The tumor sizes were traced and recorded every two days for twelve days in total.

### Chromatin immunoprecipitation (ChIP)

ChIP was performed as described previously ^49^. Briefly, samples of 60 million cells were trypsinized by washing one time with room temperature PBS, then every 50–60 million cells were resuspended in 30ml room temperature PBS. Cells were fixed with 1% formaldehyde for 8 mins at room temperature with rotation. Excessive formaldehyde was quenched with 0.25M glycine. Fixed cells were washed twice with cold PBS supplemented with 1mM PMSF. After washing with PBS, cells were lysed with ChIP SDS lysis buffer (100 mM NaCl,50 mM Tris-Cl pH8.0, 5 mM EDTA, 0.5% SDS, 0.02% NaN_3_, and fresh protease inhibitor cOmplete tablet EDTA-free (5056489001, Roche) and then stored at −80 °C until further processing. Nuclei were collected by spinning down at 3000 rpm at 4°C for 10 mins. The nuclear pellet was resuspended in IP solution (2 volume ChIP SDS lysis buffer plus 1 volume ChIP triton dilution buffer (100 mM Tris-Cl pH8.6, 100 mM NaCl, 5 mM EDTA, 5% Triton X-100), and fresh proteinase inhibitor) with 10million cells/ml IP buffer concentration for sonication using a Bioruptor (8–10 cycles, 30s on, 30s off, High power) to obtain 200 bp to 500 bp DNA fragments. After spinning down to remove debris, 1.2ml sonicated chromatin was pre-cleared by adding 50 μl washed dynabeads protein A (Thermo Scientific) and rotated at 4 °C for 2 hrs. Pre-cleared chromatin was incubated with antibody pre-bound dynabeads protein A (Thermo Scientific) overnight at 4 °C. For histone marker antibodies, 50 μl of Dynabeads protein A was loaded with 3 μg antibody. The next day, magnetic beads were washed through the following steps: buffer 1 (150 mM NaCl, 50 mM Tris-Cl, 1 mM EDTA, 5% sucrose, 0.02% NaN_3_, 1% Triton X-100, 0.2% SDS, pH 8.0) two times; buffer 2 (0.1% deoxycholic acid, 1 mM EDTA, 50 mM HEPES, 500 mM NaCl, 1% Triton X-100, 0.02% NaCl, pH 8.0) two times; buffer 3 (0.5% deoxycholic acid, 1 mM EDTA, 250 mM LiCl, 0.5% NP40, 0.02% NaN_3_) two times; TE buffer one time. To reverse crosslinks, samples were incubated with 20μg/ml proteinase K (Ambion) at 65°C overnight. The samples were then extracted with phenol:chloroform:isoamyl alcohol (25:24:1) followed by chloroform, ethanol precipitated in the presence of glycogen, and re-suspended in 10mM Tris buffer (pH 8). After reverse crosslinking and purification of DNA, qPCR was performed with the primers listed in **Supplemental Table 9**. Briefly, *p16* primer detecting the enrichments of all histone markers is located in the proximal promoter region within 100 bp around TSS; primers located 50 kb upstream of *p16* (Neg 1) and 10 kb downstream of *p16* (Neg 2) are the negative control primers located in the regions without enrichment of any of the above proteins. The antibodies used in ChIP assays were: H3K4Me3 (C42D8, #9751, Cell Signaling Technologies), H3K27Ac (ab45173, Abcam), H3K9Me3 (D4W1U, #13969, Cell Signaling Technologies), Rabbit IgG monoclonal (ab172730, Santa Cruz).

### Circularized Chromosome Conformation Capture (4C)- Seq

4C-seq was performed as described previously ^50^ with modifications ^51^. In brief, SNU-398 cells with stable CRISPR-DiR treatment for 13 days were used for 4C-Seq. 30 million sample a) guided by GN2 non-targeting and 30 million sample b) guided by guides (G19, G36, G110, and G111) targeting region LF+RF were crosslinked in 1% formaldehyde for 10 mins at RT with rotation. Then formaldehyde was neutralized by adding 2.5 M glycine to a final concentration of 0.25 M and rotating for 5 mins at RT. After washing in cold PBS, cells were resuspended in 9 ml lysis buffer (10mM Tris-HCl pH8.0, 10mM NaCl, 5mM EDTA, 0.5% NP 40, with addition of EDTA-free protease inhibitor (complete tablet, freshly dissolved in nuclease free water to make a 100X stock, 5056489001, Roche) and lysed multiple times with resuspension every 2–3 mins during the 10mins incubation on ice. After lysis, each lysate was split into two 15ml falcon tubes for Viewpoint 1 (Csp6I) or Viewpoint 2 (DpnII), respectively (4.5ml/tube, 15 million cells). After spinning down at 3,000 rpm for 10 mins at 4 °C, each nuclear preparation was washed with 500 μl 1X CutSmart buffer from NEB and spun at 800g for 10 min at 4°C, followed by resuspension into 450 μl nuclease free (NF) H_2_O and transferring exactly 450 μl of the sample into a 1.5mL eppendorf tube. To each tube, 60 μl of 10X restriction enzyme buffer provided together with the corresponding restriction enzyme (Viewpoint 1: 10X Buffer B (ER0211, Invitrogen); Viewpoint 2: 10X NEBuffer^™^ DpnII (R0543M, NEB)) and 15 μl of 10% SDS buffer were added to the 450 μl sample, followed by an incubation at 37 °C for 1 hour with shaking (900 RPM, Eppendorf Thermomixer), followed by adding 75 μl of 20% Triton X-100 to each tube for 1-hour incubation at 37 °C with shaking (900 RPM). 20 μl samples from each tube were taken out as “undigested” and stored at −20 °C. For Viewpoint 1, 50 μl Csp6I (ER0211, Invitrogen) was added (500 U per tube) together with 5.6 μl 10X Buffer B (ER0211, Invitrogen) for 18 hours digestion at 37 °C with shaking (700 RPM); for Viewpoint 2, 10 μl DpnII (R0543M, NEB) (500 U per tube) together with 8 μl NF H2O and 2 μl 10X NEBuffer^™^ DpnII were added for 18 hours digestion at 37 °C with shaking (700 RPM). The next day, after removing 20 μl of the sample for de-crosslinking, confirming a digestion efficiency over 80%, and performing PicoGreen DNA quantification to check the DNA concentration in each reaction, 10ug of the digested DNA was taken out into a new tube and the volume was adjusted to 600 μl with NF H_2_O. The samples were heat inactivated at 65 °C for 20 min. Heat inactivated chromatin was added into 1X ligation buffer (EL0013, Invitrogen) supplemented with 1% Triton X-100, 0.1 mg/ml BSA, and the volume was adjusted with NF H_2_O to 10 ml with a final DNA concentration of 1 ng/μl. After adding 660 U T4 DNA ligase (EL0013, Invitrogen 30U/μl), samples were incubated at 16 °C in thermal incubator without shaking. The next day, a final concentration of 0.5% SDS and 0.05mg/ml proteinase K (Ambion) were added to each sample, followed by 65 °C incubation overnight for de-crosslinking. The next day, after adding 30 μl of RNase A (10mg/ml, PureLink), samples were incubated at 37°C for an hour, followed by phenol: chloroform DNA purification. The chromatin was extracted with phenol:chloroform:isoamyl alcohol (25:24:1) followed by chloroform, ethanol precipitated (split to 5ml/tube and topped up with NF H_2_O to 15ml, then adding 100% ethanol to 68% to avoid SDS precipitation) in the presence of glycogen and dissolved in 10 mM Tris buffer (pH8). The ligated chromatin was analyzed by agarose gel electrophoresis and the concentration was determined by QUBIT HS DNA kit. 7 μg of ligated chromatin was digested with 10U specific second cutter NlaIII (R0125S, NEB) in 100 μl system with CutSmart Buffer (NEB), 37°C overnight without shaking. 5 μl digested chromatin was analyzed by gel electrophoresis prior to heat inactivation. Restriction enzyme was heat-inactivated by incubating the chromatin at 65°C for 20 mins. 7 μg NlaIII digested chromatin was ligated with T4 DNA ligase (EL0013, Invitrogen, 30U/μl) at 20 U/ml in 1X ligation buffer (EL0013, Invitrogen), incubated at 16°C overnight. The ligated DNA was recovered by phenol:chloroform:isoamyl alcohol (25:24:1) extraction and ethanol precipitation. 100 ng DNA of each sample was used for 4C library preparation. The library was constructed by inverse PCR and nested PCR with KAPA HiFi HotStart ReadyMix (KK2602). The 1st PCR was performed at 100 ng DNA+1.75 μl 1^st^ PCR primer mix+12.5 μl KAPA HiFi HotStart ReadyMix+H2O to 25 μl. The 1^st^ PCR program was 95°C, 3 min, 15 cycle of (98 °C, 20s; 65°C, 15s; 72 °C, 1 min), 72 °C, 5min, 4 °C hold. The 1^st^ PCR products were purified by MinElute PCR Purification Kit (28004, Qiagen) and eluted in 13 μl Elution Buffer in the kit. The 2^nd^ PCR was performed at purified 1^st^ PCR product+1.75 μl 2^nd^ PCR primer mix+12.5 μl KAPA HiFi HotStart ReadyMix+H2O to 25 μl. The 2^nd^ PCR program was 95°C, 3 min, 13 cycle of (98 °C, 20s; 65°C, 15s; 72 °C, 1 min), 72 °C, 5min, 4 °C hold. The 2^nd^ PCR products were purified by MinElute PCR Purification Kit (28004, Qiagen) and eluted in 10 μl Elution Buffer in the kit. The primer mix was 5 μl 100M forward primer + 5 μl 100M reverse primer + 90 μl H2O. All primer sequences and barcodes are listed in **Supplemental Table 10**. The libraries were subjected to size selection (250–600 bp) on a 4–20% TBE PAGE gel (Thermo Scientific). The TBE gel was run at 180V, 55 mins, stained with Sybr Safe and visualized with gel safe, and the libraries were extracted from PAGE using a gel crush protocol. Picogreen quantification, Bioanalyzer, and KAPA library quantification were performed to check the quality, size and amount of the recovered libraries, and NextSeq 500/550 Mid Output kit V2.5 (150 Cycles) (20024904, Illumina) was used for single end Nextseq sequencing.

### Statistical analysis

Methylation changes of clones analysed by bisulphite sequencing PCR (BSP) were calculated using the online methylation analysis tool QUMA (http://quma.cdb.riken.jp/). Subsequent figures and heatmaps were in R^52^ using pheatmap^53^. For mRNA qRT-PCR and ChIP-qPCR, p-values were calculated using paired/unpaired t-tests as indicated in figure legends using GraphPad Prism Software (v9.2). P <0.05 values were considered statistically significant (*P < 0.05; **P < 0.01; ***P < 0.001). All plots show mean ± SD of triplicates. Miscellaneous bar graphs and statistical tests were performed using Prism (v9.2 – 10.2.2).

### Bioinformatic analysis

#### Histone marks ChIP-Seq Analysis

The human and mouse histone mark (H3K4Me1, H3K4me2(human), H3K4Me3, H3K9ac(mouse)m H3K14ac, H3K27Ac, H3K36me3) enrichments shown in Figure 3a–d were determined by analyzing ChIP-seq data cross the cell lines A549, GM12878, H1-hESC, HCT116, HEPG2, HMEC, HSMM, HUVEC, K562, NHEK, NHLF, SNU398 obtained from ENCODE and GEO (downloaded Feb/Mar-21). Below are tables indicating which accession ids were utilized for each analysis.

#### H3K4me1 (human and mouse)

**Table:** 

	ChIP_repl1	ChIP_repl2	ChIP_repl3	Control_repl1	Control_repl2	Control_repl3	Control_repl4	Control_repl5
a549	ENCFF660AKA	ENCFF789SAF	ENCFF654DWA	ENCFF806LFR	ENCFF136DNO	ENCFF877DLC		
gm12878	ENCFF000ASM	ENCFF000ATK		ENCFF000ARK	ENCFF000ARO			
h1es	ENCFF000AXJ	ENCFF000AXM		ENCFF689EIT	ENCFF663FLW			
hct116	ENCFF000VCI	ENCFF000VCK		ENCFF000VCY	ENCFF000VCW			
hepg2	ENCFF000BEX	ENCFF000BEY		ENCFF574VLI	ENCFF642ZJC	ENCFF162ADN	ENCFF474ZPM	
hmec	ENCFF000BJD	ENCFF000BJE		ENCFFF556RIX	ENCFF788YKS	ENCFF000BHE	ENCFF749GHL	ENCFF913IHA
hsmm	ENCFF000BMA	ENCFF000BLX		ENCFF000BKK	ENCFF000BKI			
huvec	ENCFF000BTD	ENCFF000BSY		ENCFF000BQW	ENCFF000BQS	ENCFF000BQV		
k562	ENCFF000BXX	ENCFF000BYG		ENCFF000BWK	ENCFF283HQV	ENCFF994FIB	ENCFF561WFK	ENCFF156ECZ
nhek	SRR1984639	SRR1984640		SRR1984643	SRR1984644			
nhlf	ENCFF000CSM	ENCF000CSJ		ENCFF000CQP	ENCFF000CQO			
snu398								
								
B6NCrl - BM	ENCFF001JXW	ENCFF001JXV		ENCFF001JYN	ENCFF001JYM			
B6NCrl - Heart	ENCFF854EKF	ENCFF410JWH		ENCFF183PVA	ENCFF929WDB			
B6NCrl - Intestine	ENCFF566NFW	ENCFF861WQV		ENCFF879XFV	ENCFF076IRY	ENCFF856YXG	ENCFF155LYM	
B6NCrl - Kidney	ENCFF805QZD	ENCFF772KXZ		ENCFF710GYU	ENCFF292YPK	ENCFF883NHJ		
B6NCrl - Liver	ENCFF116RSC	ENCFF386JTQ	ENCFF341QIR	ENCFF803RGE	ENCFF832IQF	ENCFF167BYV		
B6NCrl - Lung	ENCFF286ZHO	ENCFF093GXT		ENCFF511ESM	ENCFF634EPK	ENCFF145WXV	ENCFF263OPF	
B6NCrl - Spleen	ENCFF001KUM	ENCFF001KUP		ENCFF001KVR	ENCFF001KWE			
C2C12								
CH12.LX	ENCFF001KAQ	ENCFF001KAR		ENCFF001KCH	ENCFF001KCI			
E14TG2a.4	ENCFF001ZGS	ENCFF001ZGU		ENCFF001ZGK	ENCFF001ZGM			
ES-Bruce4	ENCFF001KEF			ENCFF001KEU	ENCFF001KEX			
ES-e14	ENCFF001KFG			ENCFF659VUB				
G1e	ENCFF001LZG			ENCFF001MPG				
Mel	ENCFF001KPT	ENCFF001KPV		ENCFF001KRJ	ENCFF001KRK			

#### H3K4me2 (human only)

**Table:** 

	ChIP_repl1	ChIP_repl2	Chip_repl3	Control_repl1	Control_repl2	Control_repl3	Control_repl4	Control_repl5
a549	ENCFF200MJB	ENCFF144IZV	ENCFF758ZYB	ENCFF806LFR	ENCFF136DNO	ENCFF877DLC		
gm12878	ENCFF000ATO	ENCFF000ATW		ENCFF000ARK	ENCFF000ARO			
h1es	ENCFF000AXR	ENCFF000AXQ		ENCFF036EGF	ENCFF191QXK	ENCFF145GCJ	ENCFF393KUX	
hct116	ENCFF936MMN	ENCFF937OOL		ENCFF048ZOQ	ENCFF827YXC			
hepg2								
hmec	ENCFF000BJF	ENCFF000BJJ		ENCFFF556RIX	ENCFF788YKS	ENCFF000BHE	ENCFF749GHL	ENCFF913IHA
hsmm	ENCFF000BMF	ENCFF000BMI		ENCFF000BKK	ENCFF000BKI			
huvec	ENCFF000BTF	ENCFF000BTN		ENCFF000BQW	ENCFF000BQS	ENCFF000BQV		
k562	ENCFF000BYA	ENCFF000BYF		ENCFF000BWK	ENCFF283HQV	ENCFF994FIB	ENCFF561WFK	ENCFF156ECZ
nhek	ENCFF000COM	ENCFF000COT	ENCFF000COR	ENCFF000CMD				
nhlf	ENCFF000CSQ	ENCFF000CSR		ENCFF000CQP	ENCFF000CQO			
snu398								

#### H3K4me3 (human and mouse)

**Table:** 

	ChIP_repl1	ChIP_repl2	ChIP_repl3	Control_repl1	Control_repl2	Control_repl3	Control_repl4	Control_repl5
a549	ENCFF552FLH	ENCFF681VDV	ENCFF494CRS	ENCFF806LFR	ENCFF136DNO	ENCFF877DLC		
gm12878	ENCFF000AUB	ENCFF000ASR		ENCFF000ARK	ENCFF000ARO			
h1es	ENCFF000AXS	ENCFF000AXZ		ENCFF689EIT	ENCFF663FLW			
hct116	ENCFF001FIS	ENCFF001FIZ		ENCFF001HME				
hepg2	ENCFF000BGE	ENCFF000BGF		ENCFF574VLI	ENCFF642ZJC	ENCFF162ADN	ENCFF474ZPM	
hmec	ENCFF113ECY	ENCFF904QEC		ENCFFF556RIX	ENCFF788YKS	ENCFF000BHE	ENCFF749GHL	ENCFF913IHA
hsmm	ENCFF243OFA	ENCFF794IID		ENCFF000BKK	ENCFF000BKI			
huvec	ENCFF255MRT	ENCFF356SYW		ENCFF000BQW	ENCFF000BQS	ENCFF000BQV		
k562	ENCFF894KBP	ENCFF010SAE		ENCFF000BWK	ENCFF283HQV	ENCFF994FIB	ENCFF561WFK	ENCFF156ECZ
nhek	SRR1984641	SRR1984642		SRR1984643	SRR1984644			
nhlf	ENCFF238HDJ	ENCFF612NOM		ENCFF000CQP	ENCFF000CQO			
snu398								
								
B6NCrl - BM	ENCFF001JYF	ENCFF001JYG		ENCFF001JYN	ENCFF001JYM			
B6NCrl - Heart	ENCFF907BFC	ENCFF708TCP		ENCFF183PVA	ENCFF929WDB			
B6NCrl - Intestine	ENCFF439UTP	ENCFF949BVF		ENCFF879XFV	ENCFF076IRY	ENCFF856YXG	ENCFF155LYM	
B6NCrl - Kidney	ENCFF643UJF	ENCFF198CRA		ENCFF710GYU	ENCFF292YPK	ENCFF883NHJ		
B6NCrl - Liver	ENCFF589VTE	ENCFF898DBR		ENCFF803RGE	ENCFF832IQF	ENCFF167BYV		
B6NCrl - Lung	ENCFF051ZGY	ENCFF728HAD		ENCFF511ESM	ENCFF634EPK	ENCFF145WXV	ENCFF263OPF	
B6NCrl - Spleen	ENCFF001KUR	ENCFF001KUV		ENCFF001KVR	ENCFF001KWE			
C2C12	ENCFF001IBG			ENCFF001IFD	ENCFF001IFC	ENCFF001IFI		
CH12.LX	ENCFF001KAZ	ENCFF001KBF		ENCFF001KCH	ENCFF001KCI			
E14TG2a.4	ENCFF001ZOT	ENCFF001ZPA		ENCFF001ZGK	ENCFF001ZGM			
ES-Bruce4	ENCFF001KER	ENCFF001KEQ		ENCFF001KEU	ENCFF001KEX			
ES-e14	ENCFF001KFD			ENCFF659VUB				
G1e	ENCFF001LZR			ENCFF001MPG				
Mel	ENCFF001KQA	ENCFF001KQB		ENCFF001KRJ	ENCFF001KRK			

#### H3K9ac (mouse only)

**Table:** 

	ChIP_repl1	ChIP_repl2	ChIP_repl3	Control_repl1	Control_repl2	Control_repl3	Control_repl4	Control_repl5
B6NCrl - BM								
B6NCrl - Heart	ENCFF001ZSP	ENCFF001ZSM		ENCFF001ZUY	ENCFF001ZVC			
B6NCrl - Intestine	ENCFF002AOA	ENCFF002AOC		ENCFF002APX	ENCFF002AQA			
B6NCrl - Kidney	ENCFF002AOD	ENCFF002AOE		ENCFF002AOD	ENCFF002AOE			
B6NCrl - Liver	ENCFF001ZSX	ENCFF001ZSY		ENCFF001ZUT	ENCFF001ZUU			
B6NCrl - Lung	ENCFF002AOJ	ENCFF002AON		ENCFF002AQJ	ENCFF002AQI			
B6NCrl - Spleen								
C2C12								
CH12.LX	ENCFF001KBH	ENCFF001KBL		ENCFF001KCH	ENCFF001KCI			
E14TG2a.4								
ES-Bruce4	ENCFF001KDK	ENCFF001KDL		ENCFF001KEU	ENCFF001KEX			
ES-e14	ENCFF001KFS	ENCFF001KFY		ENCFF659VUB				
G1e								
Mel	ENCFF001KQN	ENCFF001KQJ		ENCFF001NCF	ENCFF001NCH			

#### H3K14ac (human)

**Table:** 

	ChIP_repl1	ChIP_repl2	Chip_repl3	Control_repl1	Control_repl2	Control_repl3	Control_repl4	Control_repl5
a549	SRR618278			SRR618277				
gm12878								
h1es	ENCFF405OWA	ENCFF951FST	ENCFF775OFR	ENCFF693FJC	ENCFF454ZHO			
hct116	SRR16991206	SRR16991207	SRR16991208	SRR16991224	SRR16991225	SRR16991226	SRR16991227	
hepg2								
hmec								
hsmm								
huvec	SRR13021978	SRR13021979	SRR13021980	SRR13021983	SRR13021984			
k562								
nhek								
nhlf								
snu398								

#### H3K27ac (human and mouse)

**Table:** 

	ChIP_repl1	ChIP_repl2	ChIP_repl3	Control_repl1	Control_repl2	Control_repl3	Control_repl4	Control_repl5
a549	ENCFF823YGI	ENCFF187VER	ENCFF573OYR	ENCFF806LFR	ENCFF136DNO	ENCFF877DLC		
gm12878	ENCFF000ASU	ENCFF000ASP		ENCFF000ARK	ENCFF000ARO			
h1es	ENCFF000AWR	ENCFF000AWV		ENCFF689EIT	ENCFF663FLW			
hct116	ENCFF000VCQ	ENCFF000VCS		ENCFF000VCY	ENCFF000VCW			
hepg2	ENCFF000BFD	ENCFF000BFH		ENCFF574VLI	ENCFF642ZJC	ENCFF162ADN	ENCFF474ZPM	
hmec	ENCFF000BIL	ENCFF000BIM		ENCFFF556RIX	ENCFF788YKS	ENCFF000BHE	ENCFF749GHL	ENCFF913IHA
hsmm	ENCFF000BLI	ENCFF000BLJ		ENCFF000BKK	ENCFF000BKI			
huvec	ENCFF000BSI	ENCFF000BSD		ENCFF000BQW	ENCFF000BQS	ENCFF000BQV		
k562	ENCFF000BXH	ENCFF000BXG		ENCFF000BWK	ENCFF283HQV	ENCFF994FIB	ENCFF561WFK	ENCFF156ECZ
nhek	SRR1984645	SRR1984646		SRR1984643	SRR1984644			
nhlf	ENCFF000CRV	ENCFF000CRS		ENCFF000CQP	ENCFF000CQO			
snu398								
								
B6NCrl - BM	ENCFF001JYA	ENCFF001JYB		ENCFF001JYN	ENCFF001JYM			
B6NCrl - Heart	ENCFF483VRP	ENCFF848QQS		ENCFF183PVA	ENCFF929WDB			
B6NCrl - Intestine	ENCFF129PWT	ENCFF183MBY		ENCFF879XFV	ENCFF076IRY	ENCFF856YXG	ENCFF155LYM	
B6NCrl - Kidney	ENCFF107WXS	ENCFF594YET		ENCFF710GYU	ENCFF292YPK	ENCFF883NHJ		
B6NCrl - Liver	ENCFF833RGU	ENCFF716OXJ		ENCFF803RGE	ENCFF832IQF	ENCFF167BYV		
B6NCrl - Lung	ENCFF176UWV	ENCFF821LPZ		ENCFF511ESM	ENCFF634EPK	ENCFF145WXV	ENCFF263OPF	
B6NCrl - Spleen	ENCFF001KUS	ENCFF001KUW		ENCFF001KVR	ENCFF001KWE			
C2C12								
CH12.LX	ENCFF001KBR	ENCFF001KBQ		ENCFF001KCH	ENCFF001KCI			
E14TG2a.4								
ES-Bruce4	ENCFF001KDQ	ENCFF001KDO		ENCFF001KEU	ENCFF001KEX			
ES-e14	ENCFF001KFR			ENCFF659VUB				
G1e								
Mel	ENCFF001KQK	ENCFF001KQL		ENCFF001KRJ	ENCFF001KRK			

#### H3K36me3 (human and mouse)

**Table:** 

	ChIP_repl1	ChIP_repl2	Chip_repl3	Control_repl1	Control_repl2	Control_repl3	Control_repl4	Control_repl5
a549	SRR618286			SRR618285				
gm12878	ENCFF000ATN	ENCFF000ATJ		ENCFF000ARK	ENCFF000ARO			
h1es	ENCFF034AQJ	ENCFF759KCC		ENCFF036EGF	ENCFF191QXK	ENCFF145GCJ	ENCFF393KUX	
hct116	ENCFF312RKB	ENCFF850EAH		ENCFF048ZOQ	ENCFF827YXC			
hepg2	ENCFF000BFT	ENCFF000BFZ	ENCFF401IDR	ENCFF356QYZ	ENCFF854ELY	ENCFF881CNC		
hmec	ENCFF000BIS	ENCFF000BIY		ENCFFF556RIX	ENCFF788YKS	ENCFF000BHE	ENCFF749GHL	ENCFF913IHA
hsmm	ENCFF000BLZ	ENCFF000BLU		ENCFF000BKK	ENCFF000BKI			
huvec	ENCFF000BSQ	ENCFF000BSS	ENCFF000BSJ	ENCFF000BQW	ENCFF000BQS	ENCFF000BQV		
k562	ENCFF000BXR	ENCFF000BXO	ENCFF544IFT	ENCFF000BWK	ENCFF283HQV	ENCFF994FIB	ENCFF561WFK	ENCFF156ECZ
nhek	ENCFF000COD	ENCFF000CNV	ENCFF000COB	ENCFF000CMD				
nhlf	ENCFF000CSE	ENCFF000CSF		ENCFF000CQP	ENCFF000CQO			
snu398								
								
B6NCrl - BM								
B6NCrl - Heart	ENCFF827ZPT	ENCFF964GOM	ENCFF337AOQ	ENCFF183PVA	ENCFF929WDB			
B6NCrl - Intestine	ENCFF946FVI	ENCFF910TMA	ENCFF994JBM	ENCFF879XFV	ENCFF076IRY	ENCFF856YXG	ENCFF155LYM	
B6NCrl - Kidney	ENCFF982KIO	ENCFF192DLE	ENCFF036EET	ENCFF710GYU	ENCFF292YPK	ENCFF883NHJ		
B6NCrl - Liver	ENCFF547KWQ	ENCFF638IQR		ENCFF803RGE	ENCFF832IQF	ENCFF167BYV		
B6NCrl - Lung	ENCFF121EQW	ENCFF206FJL	ENCFF567IST	ENCFF511ESM	ENCFF634EPK	ENCFF145WXV	ENCFF263OPF	
B6NCrl - Spleen	ENCFF001KVK	ENCFF001KVI		ENCFF001KVR	ENCFF001KWE			
C2C12	ENCFF001IBZ			ENCFF001IFD	ENCFF001IFC	ENCFF001IFI		
CH12.LX	ENCFF001KBU	ENCFF001KBV		ENCFF001KCH	ENCFF001KCI			
E14TG2a.4	ENCFF001ZHO	ENCFF001ZHQ		ENCFF001ZGK	ENCFF001ZGM			
ES-Bruce4	ENCFF001KEE	ENCFF001KEI		ENCFF001KEU	ENCFF001KEX			
ES-e14	ENCFF001KGA	ENCFF001KGE		ENCFF659VUB				
G1e	ENCFF001MAV	ENCFF001MAU		ENCFF001MPG				
Mel	ENCFF001KQZ	ENCFF001KRA		ENCFF001KRJ	ENCFF001KRK			

The ChIP-Seq datasets were processed using ChIP-AP v5.1^54^ using the following settings tables for human and mouse respectively. Called peaks were then overlapped with identified Mesa coordinates using GenomicRanges ^55^. Only strict overlaps were accepted (at least 1bp overlap between the Mesa and the called peak).

#### Human Settings Table (ChIP-AP)

**Table:** 

program	argument
fastqc1	
clumpify	dedupe spany addcount
bbduk	ktrim=l hdist=2
trimmomatic	LEADING:20 SLIDINGWINDOW:4:20 TRAILING:20 MINLEN:20
fastqc2	
bwa_mem	
samtools_view	
plotfingerprint	
fastqc3	
macs2_callpeak	
gem	-Xmx36G –k_min 8 –k_max 12
sicer2	
homer_findpeaks	
genrich	--adjustp -v
homer_mergePeaks	
homer_annotatePeaks	
fold_change_calculator	--normfactor uniquely_mapped

#### Mouse Settings Table (ChIP-AP)

**Table:** 

program	argument
fastqc1	
clumpify	dedupe spany addcount qout=33 fixjunk
bbduk	ktrim=r hdist=21 mink=8 hdist=2 hdist2=1
trimmomatic	LEADING:20 SLIDINGWINDOW:4:20 TRAILING:20 MINLEN:20
fastqc2	-q
bwa_mem	
samtools_view	-q 20
plotfingerprint	
fastqc3	
macs2_callpeak	
gem	-Xmx10G –k_min 8 –k_max 12
sicer2	
homer_findpeaks	
genrich	--adjustp -v
homer_mergePeaks	
homer_annotatePeaks	
fold_change_calculator	--normfactor uniquely_mapped

#### WGBS and MM analysis

Raw WGBS sequenced fastq files were first processed with MSuite (v2)^56^ being aligned to hg38 using default parameters for MSuite. Custom scripts were then used to convert MSuite output to input into Defiant ^57^ and Metilene ^58^ for methylation percentage calling. Defiant was run with the flags “-c 0,2,1 -d 1,3,1 -G147 -CpN5 -p 0.05. Metilene was run with flags “-m 3 -d 0.1 -M 147 -f 1”. A custom R script was then used to merge all outputs, annotations and calculate the average methylation percentages in all regions of interest. For MM gene annotations, HOMER^59^ was used for either hg38 or mm9. CpG Island annotations and gene definitions were provided by the annotatr ^60^ R package from Bioconductor, which in turn uses the AnnotationHub package ^61^. All figures were generated in R (v4.1.2) ^52^ using the packages pheatmap ^53^, ggplot2 ^62^, UpsetR ^63^, and Sushi ^64^.

#### 4C-Seq Analysis

For the 4C-seq analysis, the long-range genomic interaction regions generated by the 4C-Seq experiment were processed using the CSI NGS portal ^65^. Briefly, raw fq files were aligned to a masked hg19 reference (masked for the gap, repetitive and ambiguous sequences) using bwa mem ^66^. Bam files were converted to read coverage files by bedtools genomecov ^67^. The read coverage was normalized according to the sequencing depth. BedGraph files of the aligned bams were converted to bigWig format by bedGraphToBigWig. Next, the processed alignment files were analyzed using r3CSeq ^68^ and using the associated masked hg19 genome (BSgenome.Hsapiens.UCSC.hg19.masked) ^69^, from the R Bioconductor repository. Chromosome 9 was selected as the viewpoint, and Csp6I, DpnII were used as the restriction enzyme to digest the genome. Smoothed bam coverage maps were generate using bamCoverage from the deeptools suite ^70^ with the flags “--normalizeUsing RPGC --binSize 2000 --smoothLength 6000 --effectiveGenomeSize 2864785220 --outFileFormat bedgraph” and plotted using the Bioconductor package Sushi ^64^ to get the viewpoint coverage depth maps. BigInteract files for UCSC and bedpe files were manually generated with the “score” values being calculated as −log( interaction_q-value_from_r3CSeq + 1*10^−10^ ). Sushi was then used to plot the bedpe files to get the 4C looping plots. To identify differential interaction peaks, HOMER’s ^71^ get DifferentialPeaks was used with the flag “-F 1.5” after which the corresponding bigInteract and bedpe files were generated as described.

## Supplementary Material

Supplement 1

## Data Availability

Sequencing data has been deposited in Gene Expression Omnibus (GSE153563). All other data is available in the main text or the supplemental materials. Reviewer access token for the GEO Submission is **ovgjoisudxabbmp**. For manuscript review only, reviewers may view our UCSC track hubs for the WGBS data using the following session URL – https://genome.ucsc.edu/s/mbassal/mesa_review_hg38
