# Supplementary material for: Methylation Mesa define functional regulatory elements for targeted gene activation
Source: Res Sq. 2024 Oct 16:rs.3.rs-4359582. Preprint. [Version 1] doi: 10.21203/rs.3.rs-4359582/v1 (PMC11527235; doi:10.21203/rs.3.rs-4359582/v1)
Supplement: Supplement 1 [file NIHPPrs4359582v1-supplement-1.pdf]

Supplemental Figure 1

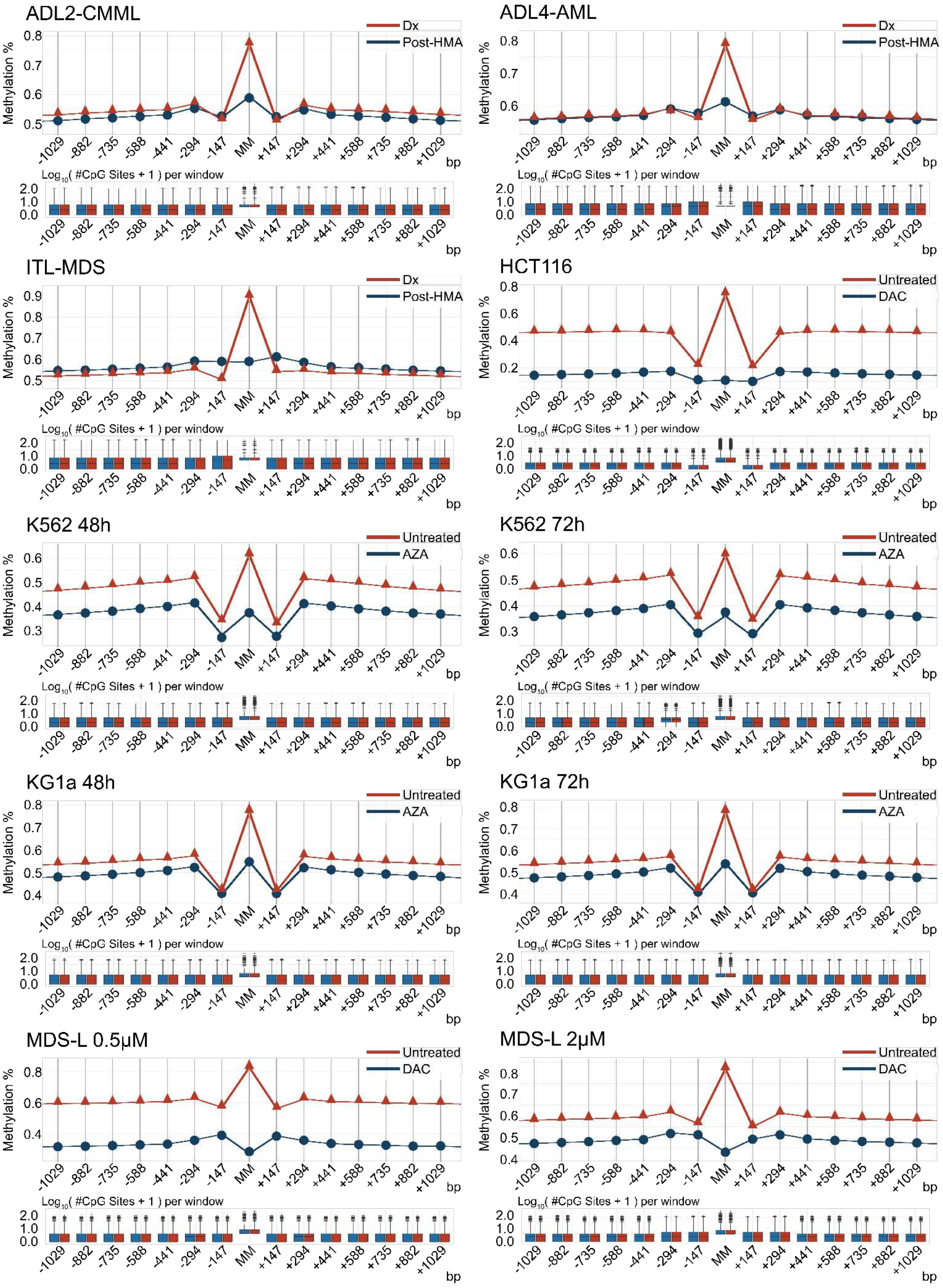

083

Supplemental Figure 1

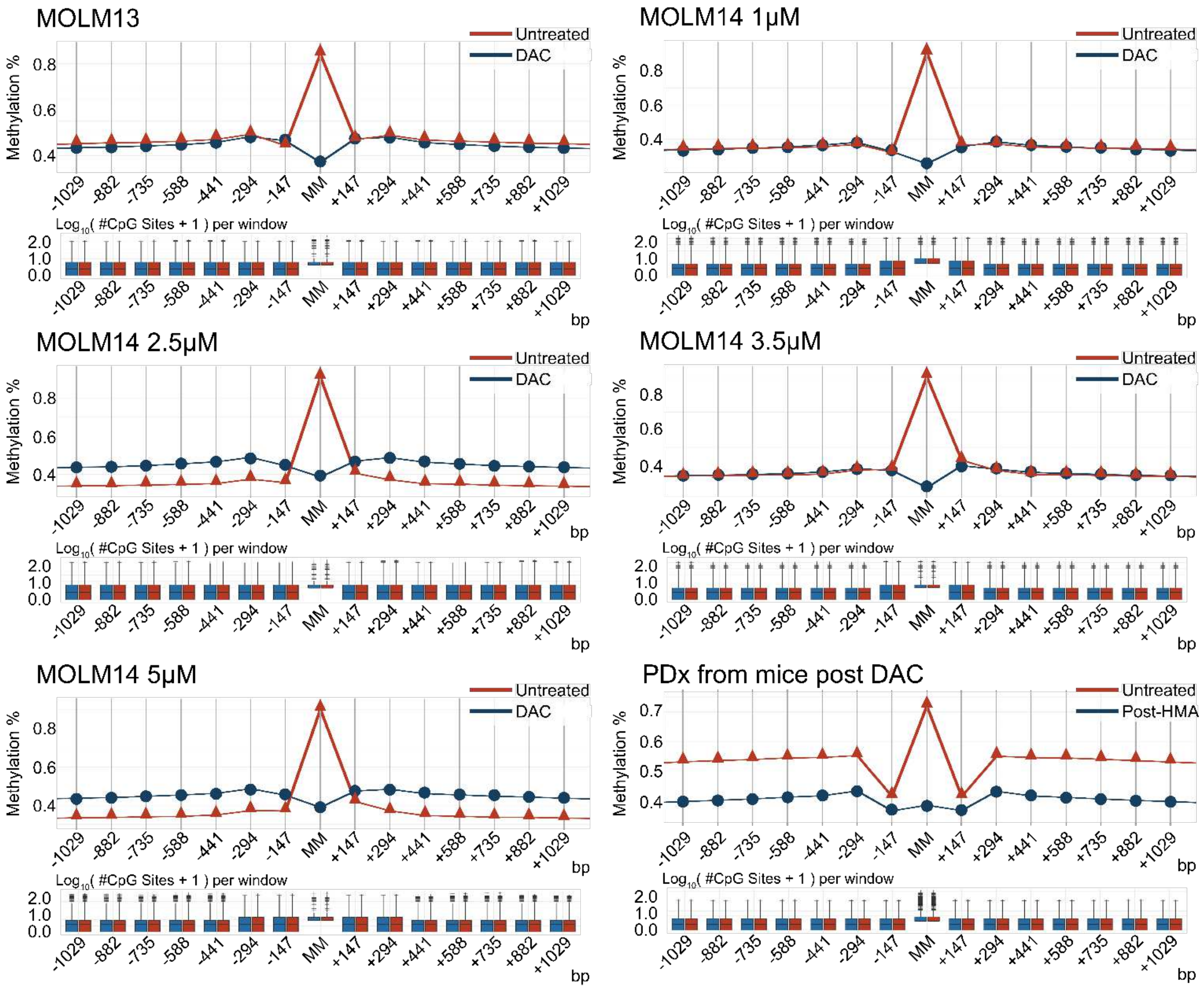

084

Supplemental Figure 1. The genomic distribution of MM classified by common annotation groups.

Average methylation profiles for untreated and HMA-treated samples showing Mesa and flanking window methylation percentages. Below the methylation profiles are matched boxplots depicting the distribution of the number of CpG sites found per corresponding window. The Y-axis shows methylation value ranging from 0 (no methylation, 0%) to 1 (methylated, 100%). The X-axis shows the MM in the center, and extending bidirectionally from each MM are the flanking windows, each 147bp in length, denoted by their relative base pair distance away from the Mesa. Within each group along the X-axis, the profiles plotted show the average methylation value across all sites identified genome-wide within that window distance away from the Mesa. The profiles corresponding to untreated/naïve samples are shown in red. The profiles corresponding to post-HMA treatment are shown in blue. Below each average methylation profile are box plots summarising the distribution of the CpG sites ($\log_{10}$ (# CpG's +1)) profiled within that window. Red box plots represent summaries of the number of CpGs found in the untreated/naïve samples, while the blue box plots show summaries of the number of CpG sites profiled in the HMA-treated sample.

Supplemental Figure 2

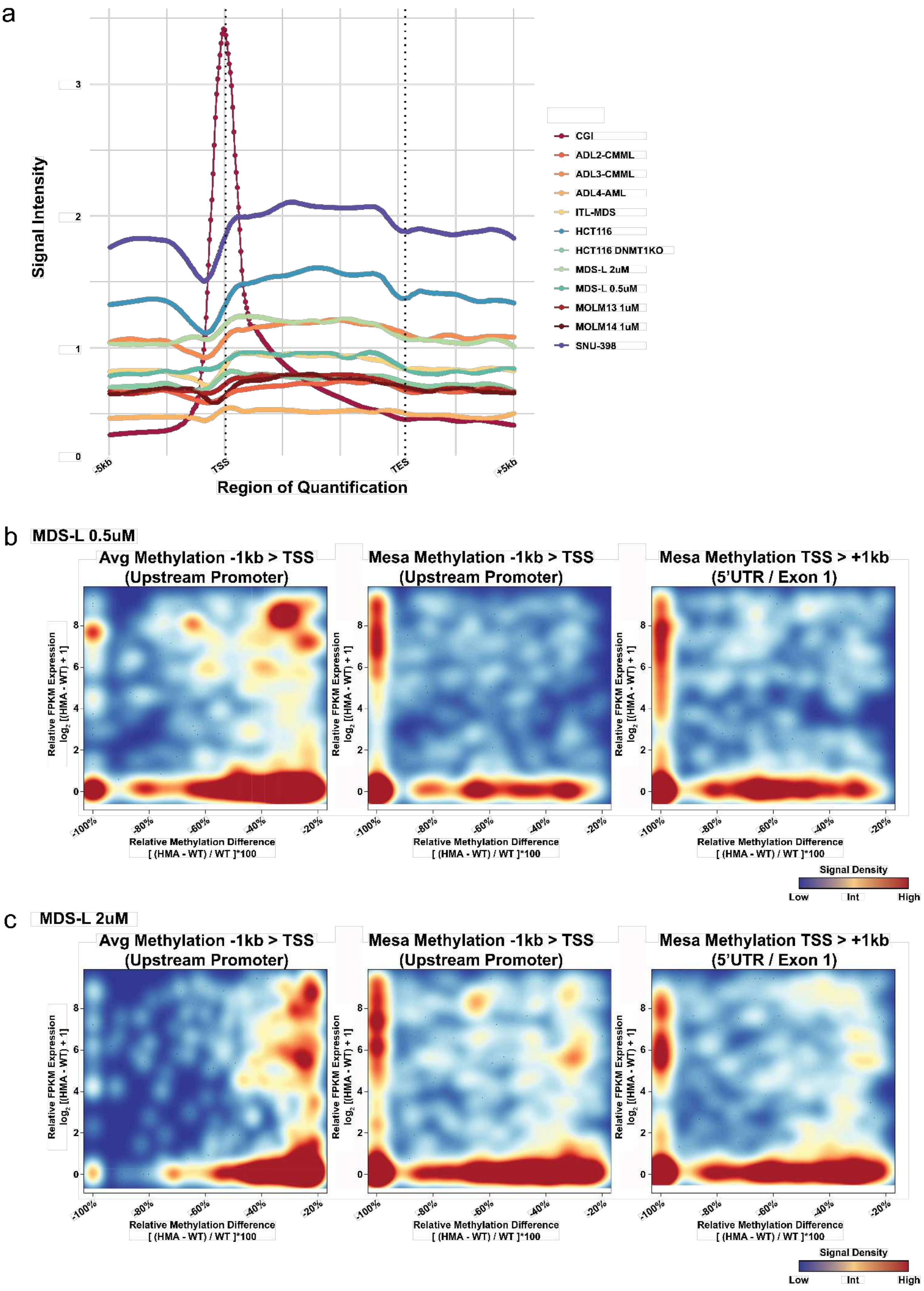

139

d MOLM14 2.5uM

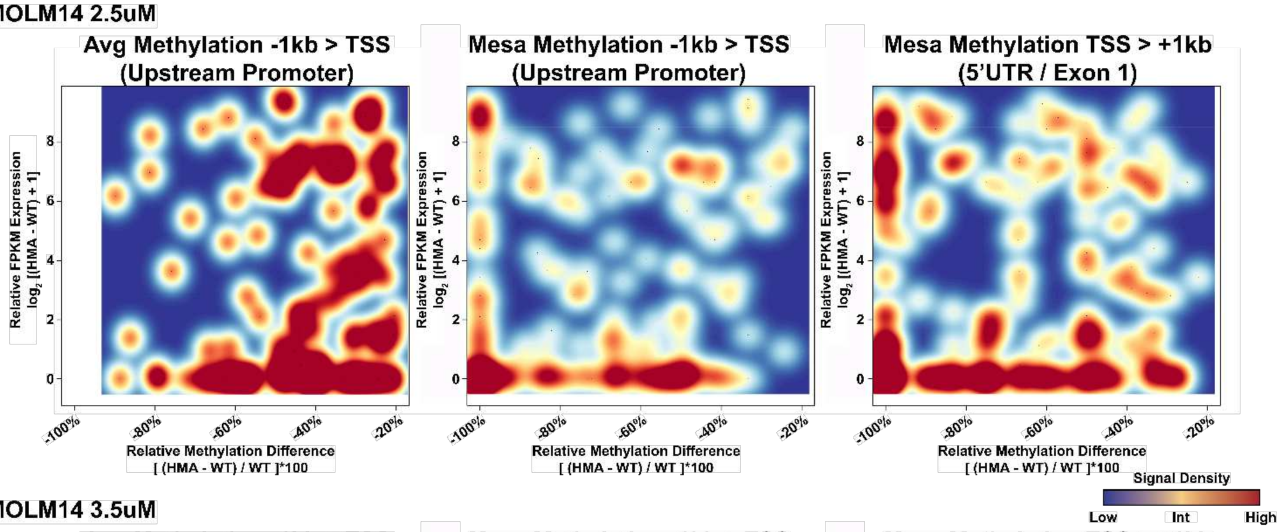

e MOLM14 3.5uM

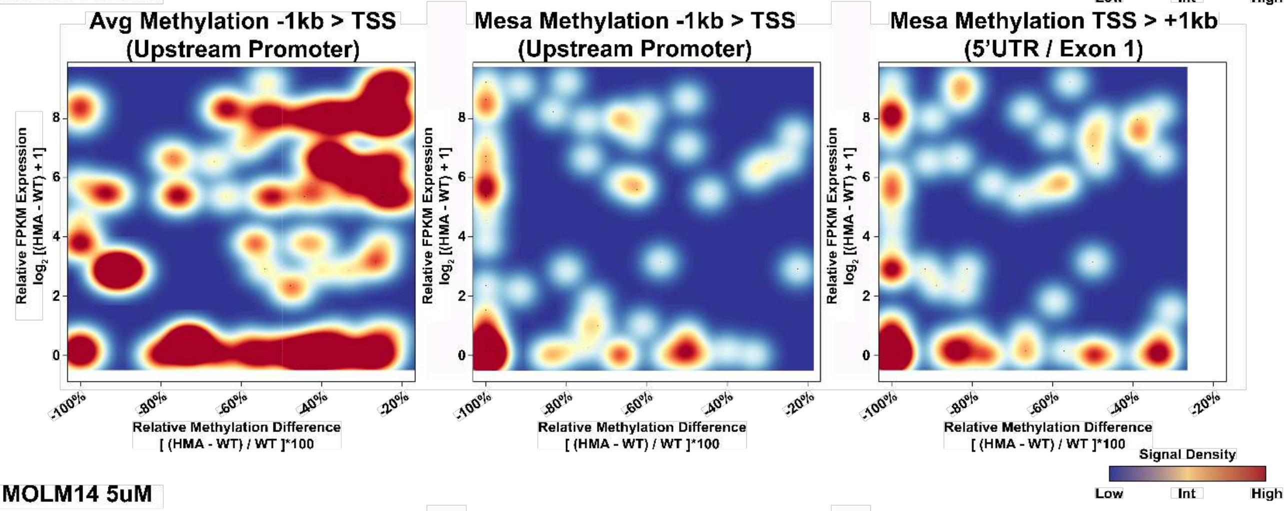

f MOLM14 5uM

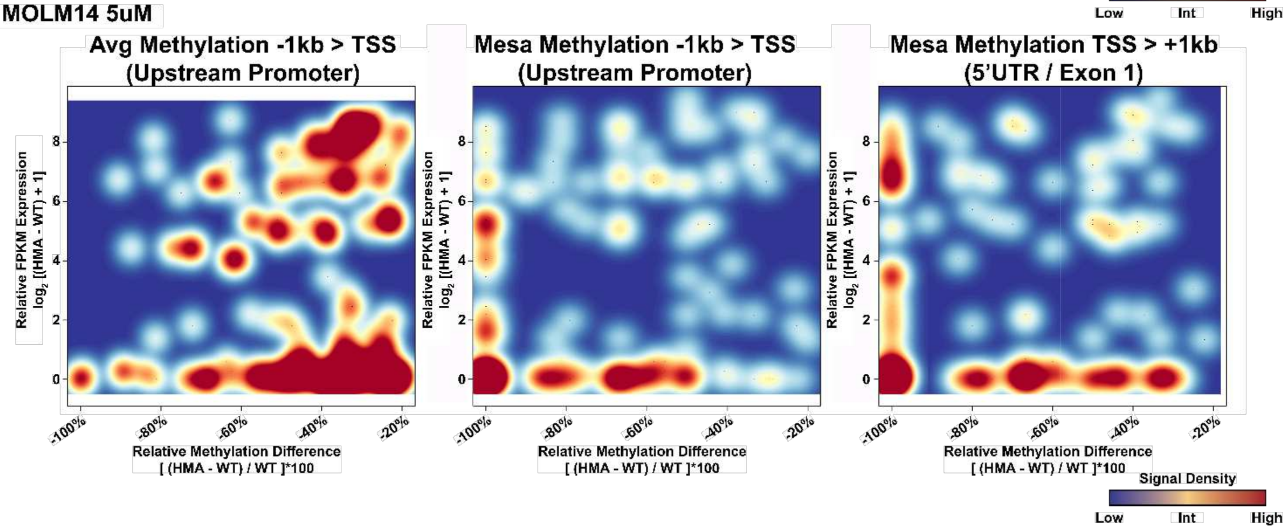

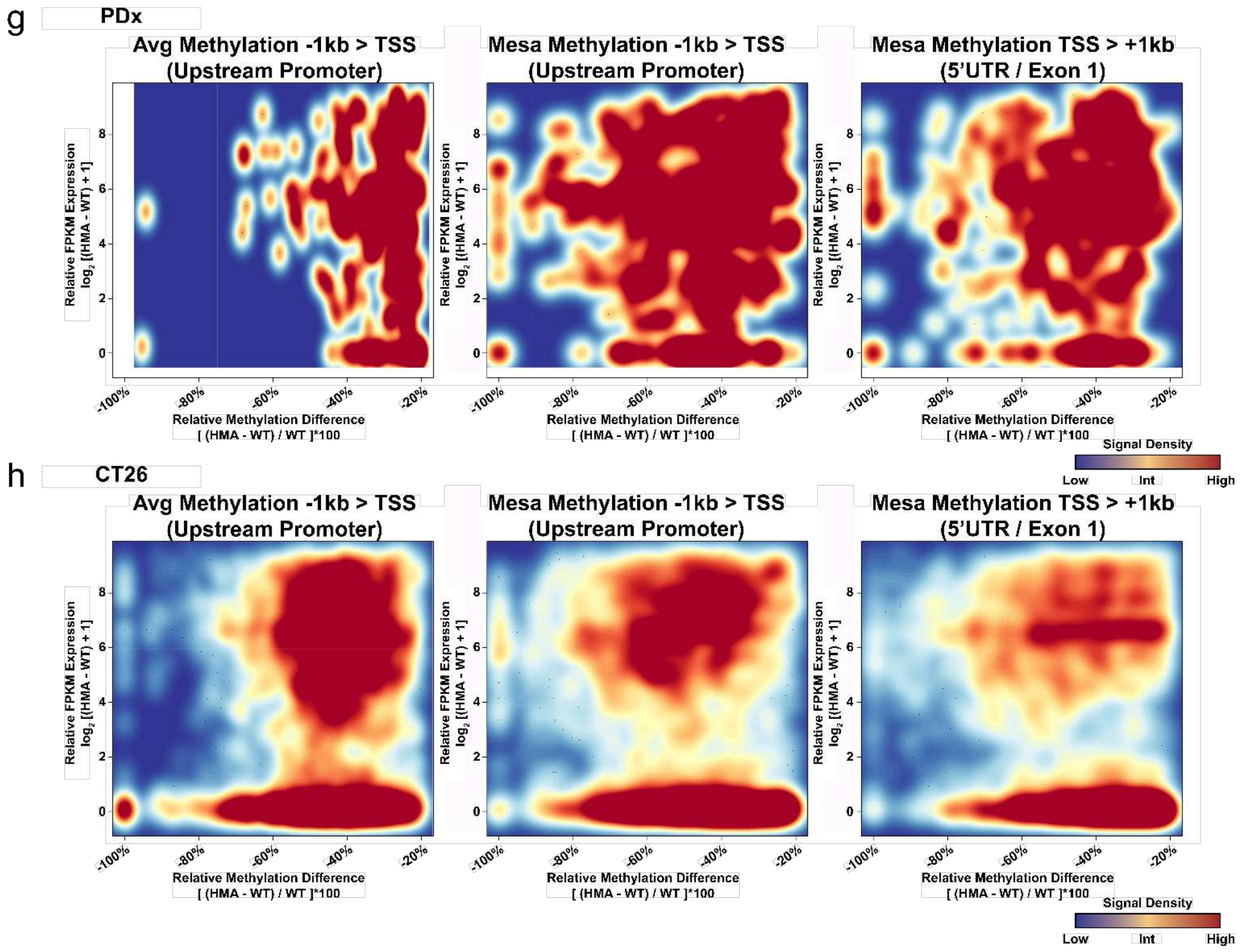

g
PDx
Avg Methylation -1kb > TSS (Upstream Promoter)
Mesa Methylation -1kb > TSS (Upstream Promoter)
Mesa Methylation TSS > +1kb (5'UTR / Exon 1)
Relative FPKM Expression
log2 [(HMA - WT) + 1]
Relative Methylation Difference
[(HMA - WT) / WT]*100
-100%
-80%
-60%
-40%
-20%
Signal Density
Low
Int
High
h
CT26
Avg Methylation -1kb > TSS (Upstream Promoter)
Mesa Methylation -1kb > TSS (Upstream Promoter)
Mesa Methylation TSS > +1kb (5'UTR / Exon 1)

141

Supplemental Figure 2. MM signal distribution and the association between Mesa methylation level with expression

(**a**) Profile plot showing presence of signal surrounding coding genes for all profiled samples. Along the region of -5kb > TSS > TES > +5kb surrounding all coding genes, 50bp bins were created and the presence (signal) of a CGI or Mesa was calculated. The profile plot shows the signal across all 50bp bins with a loess smoothing filter applied. The profile presented shows peak CGI signal just upstream of gene TSS, while Mesa elements conversely associate with CGI and instead peak within gene bodies (enriched in 5'UTRs and introns as shown in **Figure 2b**). (**b-h**) Density plots showing the association between methylation and mRNA expression. Only genes for which there was methylation and mRNA signal across all 3 compartments were utilized so as to provide consistent comparison. The X-axis shows the relative methylation difference percent between HMA-treatment and untreated/naïve (i.e., what percent does the methylation drop in the HMA-treated sample as compared to the methylation level observed in the untreated/naïve sample). The Y-axis shows the relative FPKM expression difference between HMA-treatment and untreated/naïve (i.e., how much does the expression change in the HMA-treated sample with respect to the untreated/naïve sample). Within each sub-panel, the left most density plot shows the association between average methylation across the entire 1kb promoter upstream of a TSS and mRNA expression. Signal density shows many genes with low relative methylation difference (i.e., there is little methylation difference between HMA-treatment and untreated/naïve) yet still have high relative expression, indicating a weak association between methylation level and expression. Within each sub-panel, the middle density plot shows the association between average methylation of just Mesa loci within the 1kb promoter upstream of a TSS and mRNA expression. Signal density shows (along the X-axis), genes that are undergoing demethylation (demethylating from -20 > -100%) but have still not shown expression (y=0) with many genes showing increases in relative expression only after achieving near -100% relative methylation drop in the HMA-treated sample as compared to the untreated/naïve. Within each sub-panel, the right most density plot shows the association between average methylation of just Mesa loci located within the first 1kb of a gene body (i.e., covering a gene's 5'UTR and exon 1) and mRNA expression. Signal density shows

(along the X-axis), genes that are undergoing demethylation (demethylating from -20 > -100%) but have still not shown expression (y=0) with most genes showing increases in relative expression only after achieving near -100% relative methylation drop in the HMA-treated sample as compared to the untreated/naïve. Each panel shows the following: (b) the density plots derived from the MDS-L line treated with 0.5μM of DAC, (c) the density plots derived from the MDS-L line treated with 2μM of DAC, (d) the density plots derived from the MOLM14 line treated with 2.5μM of DAC, (e) the density plots derived from the MOLM14 line treated with 3.5μM of DAC, (f) the density plots derived from the MOLM14 line treated with 5μM of DAC, (g) the density plots derived from the publicly available PDx sample analysed. Although the associations between methylation and expression observed in this sample are weaker than those observed in the human cell lines, there is still a stronger association when comparing Mesa methylation with expression than as compared to relying solely on the upstream promoter methylation. (h) The density plots derived from the publicly available CT26 mouse line analyzed. Although the associations between methylation and expression observed in this sample are weaker than those observed in the human cell lines, the strongest association between methylation and expression is still observed when associating Mesa methylation within the first 1kb of the gene body, as compared to average or Mesa methylation upstream of the TSS.

Supplemental Figure 3

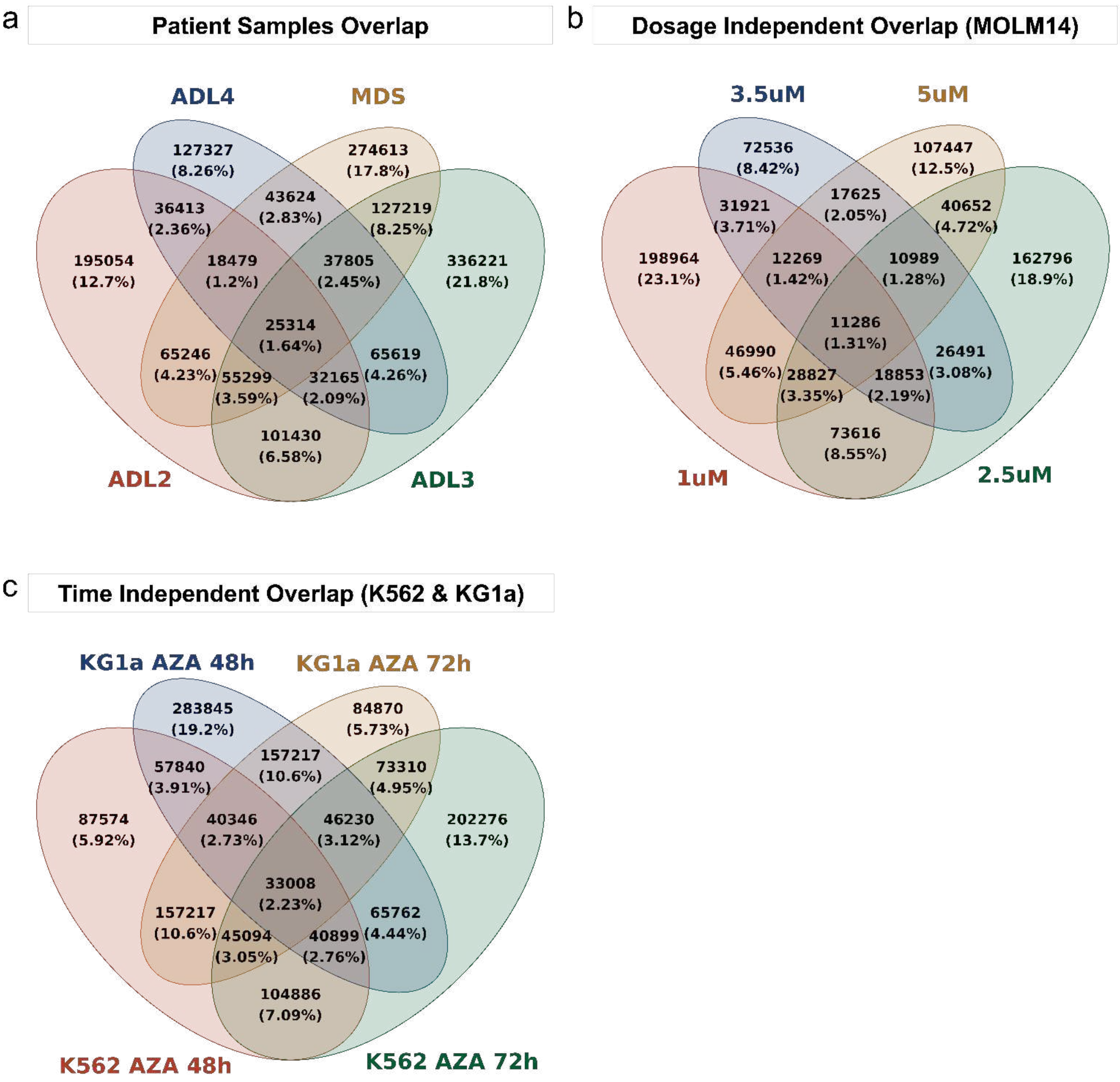

Supplemental Figure 3. MM concordance across samples independent of treatment concentration or duration

(**a**) Venn diagram showing consistent Mesa positions across highly heterogenous patient derived bone marrow samples. Mesa genomic coordinates were compared across all patient derived samples to assess consistency of identified Mesa locations. A high number of Mesa sites (~608,000) showed overlap in at least 2 patients. (**b**) Venn diagram showing consistent Mesa positions identifiable independent of HMA drug dosage used. Mesa genomic coordinates were compared across the differing DAC dosages to assess consistency of Mesa sites being triggered by HMA. Approximately 320,000 loci were found genome-wide in at least 2 DAC concentrations. (**c**) Venn diagram showing consistent Mesa positions identifiable independent of HMA treatment duration. Mesa genomic coordinates were compared across 48 and 72hr HMA treated K562 and KG1a lines to assess consistency of Mesa sites being triggered over time. Approximately 820,000 Mesa sites were found genome-wide in at least 2 time points.

Supplemental Figure 4

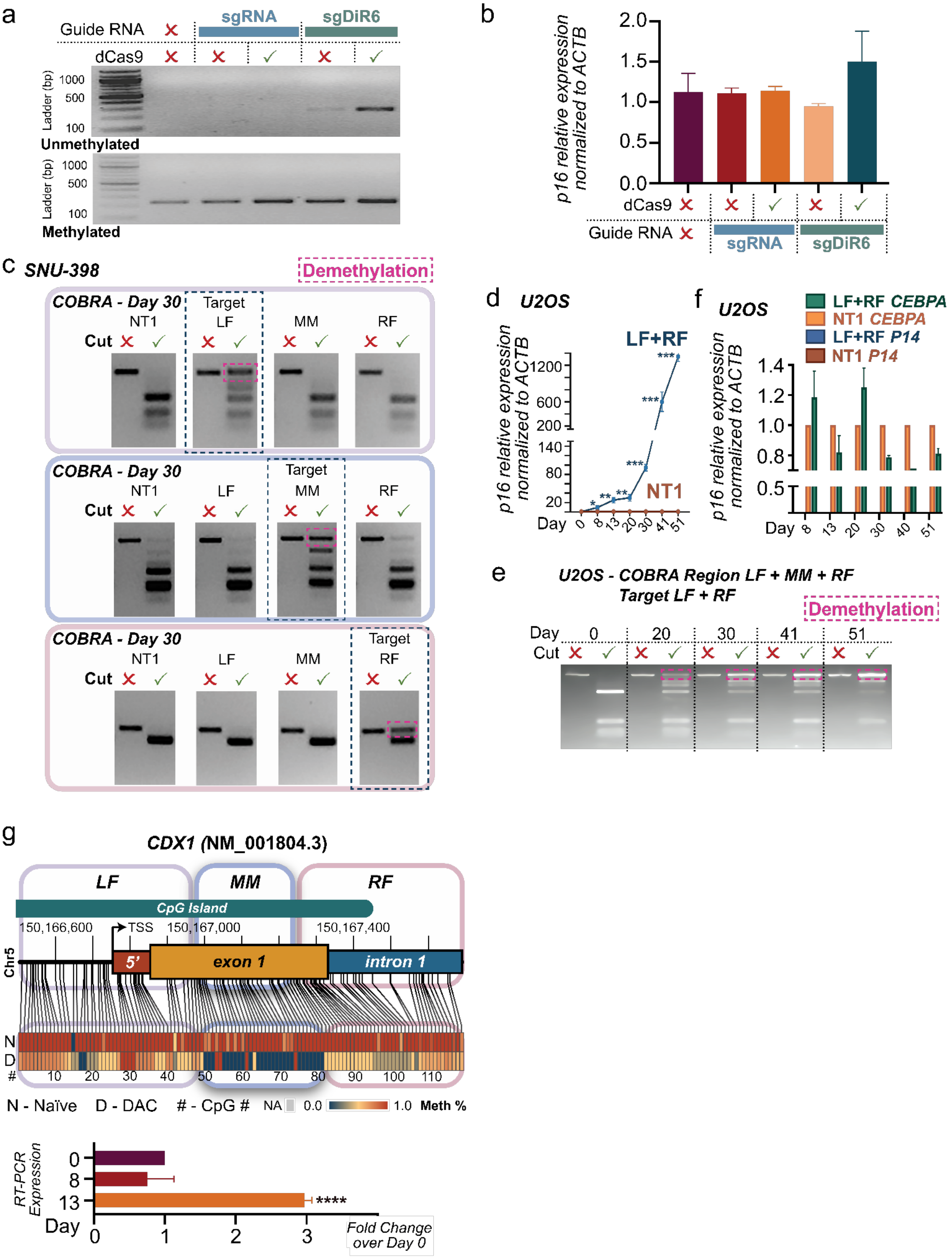

Supplemental Figure 4. Development of CRISPR-DiR and MM causal role validation across gene loci and cell types

(**a**) Methylation Sensitive PCR (MSP) data showing the *p16* demethylation in SNU398 cell line 72 h post-transfection. dCas9: dead Cas9, sgRNA: guide G1 followed by standard sgRNA scaffold (no DiR), sgDiR6: guide G1 followed by the optimal sgDiR scaffold (DiR loop R2 and R5 fused to sgRNA scaffold). (**b**) Real Time-Quantitative PCR (RT-qPCR) result showing *p16* gene expression in SNU398 cells 72 h post transfection. Mean ± SD, n = 3, *P < 0.05; **P < 0.01; ***P < 0.001. (**c**) CRISPR-DiR induced demethylation of p16 only occurs in the targeted region, with high-resolution demethylation window. The methylation in LF, MM, RF regions were detected by Combined Bisulfite Restriction Analysis (COBRA) separately at Day 30 after CRISPR-DiR non-targeting (NT1) control, or targeting LF (top panel), MM (middle panel), or RF (bottom panel). The band after cutting remain equal to that of the uncut band represents demethylated DNA. (**d-e**) p16 (**d**) mRNA assayed by RT-qPCR and (**e**) demethylation assayed by Combined Bisulfite Restriction Analysis (COBRA) in CRISPR-DiR treated human osteosarcoma U2OS cell line. Statistical significance was determined using one-tailed paired t-tests. (**f**) Real Time-Quantitative PCR (RT-qPCR) result showing the expression change of gene *p14* and gene *CEBPA* during the 51-day period of CRISPR-DiR targeting *p16* LF+RF in U2OS cells. *p14* is hypermethylated and silenced (undetectable) in U2OS, while *CEBPA* is not hypermethylated but expressed in U2OS. (**g**) Top: LF, MM, and RF region definitions. MM is calculated by WGBS data of SNU398 naïve (N) and DAC (D) treated cells. Heatmap represent the WGBS results in the corresponding loci. Bottom: Real Time-Quantitative PCR (RT-qPCR) results showing gene mRNA expression over 2 weeks in SNU398 after CRISPR-DiR targeting LF+RF of the corresponding gene. All statistics calculated using unpaired one-tailed t-tests. All panels show mean ± SD, n = 3, *P < 0.05; **P < 0.01; ***P < 0.001.

Supplemental Figure 5

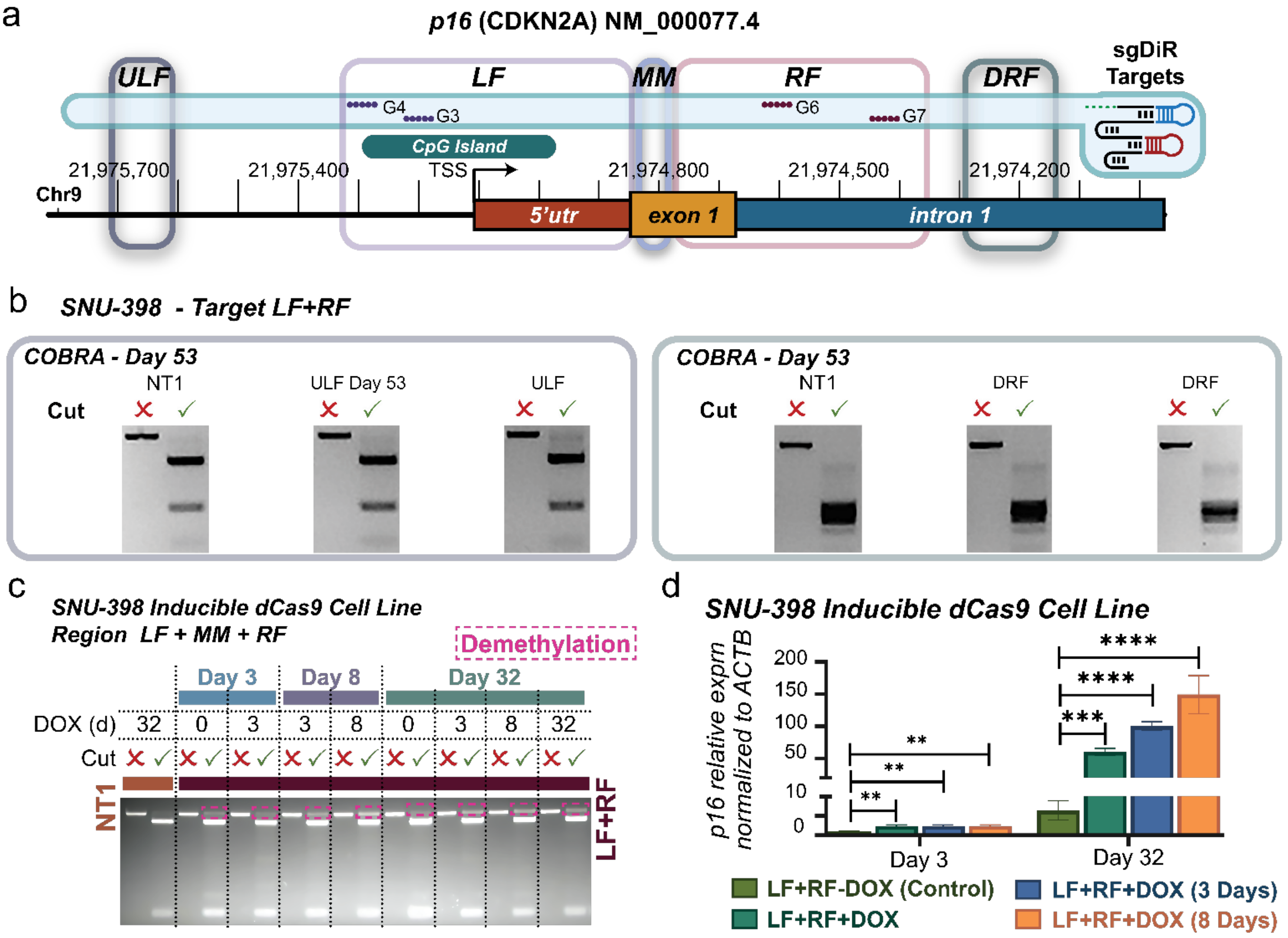

282

Supplemental Figure 5. CRISPR-DiR initiated MM demethylation does not spread, stays within boundaries, and transient demethylation effects are maintained over time

(**a**) Schematic representation of the locations of Upper Left Flank (ULF), LF, MM, RF, Downstream Right Flank (DRF). Guide G3, G4, G6, G7 were used to target p16 LF+RF when assessing methylation in ULF and DRF. (**b)** COBRA analysis of the demethylation profile in *p16* ULF and DRF at Day 53 after CRISPR-DiR non-targeting (NT1) control, or targeting LF+RF. No demethylation was observed in ULF and DRF, indicating the demethylation of LF-MM-RF would not spread out of the LF and RF boundaries. (**c**) COBRA analysis representing the demethylation profile of *p16* in inducible CRISPR-DiR SNU398 cells, the expression of dCas9 can be induced by doxycycline. The demethylation status was maintained for more than a month with as short as three days or eight days induction of CRISPR-DiR. All treatments were cultured and assayed at Day 0, Day 3, Day 8 or Day 32. All COBRA primers and restriction enzymes can be found in **Supplemental Table 8**. (**d**) RT-qPCR result showing *p16* mRNA expression for more than a month in inducible CRISPR-DiR SNU398 cells. In the inducible system, the same targeting strategy (LF+RF) was used, and dCas9 expression was induced for 0 days, 3 days, 8 days, or 32 days following treatment with Deoxycytidine (Dox). All treatments were cultured and assayed at Day 3, and Day 32. Statistical significance was calculated using one-tailed unpaired t-tests. Mean ± SD, n = 3, *P < 0.05; **P < 0.01; ***P < 0.001.

Tables

Supplemental Table 1. MM Locations and Annotations for SNU398

Supplemental Table 2. MM Locations and Annotations for HCT116

Supplemental Table 3. The sequences of sgRNA and sgDiR1-8

Supplemental Table 4. The guide RNA sequences

Supplemental Table 5. Plasmids used in transient transfection and lentivirus generated stable lines

Supplemental Table 6. The location of Region ULF, Region LF, Region MM, Region RF and Region DRF

Supplemental Table 7. Primer sequences used for qRT-PCR

Supplemental Table 8. Primers and restriction enzymes for methylation assays

Supplemental Table 9. Primer sequences for ChIP-qPCR

Supplemental Table 10. Primer sequences for 4C-Seq
